# Synthesis, Molecular Docking, and Biological Evaluation of Novel Anthranilic Acid Hybrid and Its Diamides as Antispasmodics

**DOI:** 10.3390/ijms241813855

**Published:** 2023-09-08

**Authors:** Miglena Milusheva, Vera Gledacheva, Iliyana Stefanova, Mehran Feizi-Dehnayebi, Rositsa Mihaylova, Paraskev Nedialkov, Emiliya Cherneva, Yulian Tumbarski, Slava Tsoneva, Mina Todorova, Stoyanka Nikolova

**Affiliations:** 1 Department of Organic Chemistry, Faculty of Chemistry, University of Plovdiv, 4000 Plovdiv, Bulgaria or miglena.milusheva@mu-plovdiv.bg (M.M.); minatodorova@uni-plovdiv.bg (M.T.); 2 Department of Bioorganic Chemistry, Faculty of Pharmacy, Medical University of Plovdiv, 4002 Plovdiv, Bulgaria; 3 Department of Medical Physics and Biophysics, Faculty of Pharmacy, Medical University of Plovdiv, 4002 Plovdiv, Bulgaria; vera.gledacheva@mu-plovdiv.bg (V.G.); iliyana.stefanova@mu-plovdiv.bg (I.S.); 4 Department of Chemistry, Faculty of Science, University of Sistan and Baluchestan, Zahedan P.O. Box 98135-674, Iran; m.feizi@sutech.ac.ir; 5 Laboratory of Experimental Chemotherapy, Department “Pharmacology, Pharmacotherapy and Toxicology”, Faculty of Pharmacy, Medical University, 1431 Sofia, Bulgaria; 6 Department of Pharmacognosy, Faculty of Pharmacy, Medical University of Sofia, 1000 Sofia, Bulgaria; 7 Department of Chemistry, Faculty of Pharmacy, Medical University of Sofia, 2 Dunav Str., 1000 Sofia, Bulgaria; 8 Institute of Organic Chemistry with Centre of Phytochemistry, Bulgarian Academy of Sciences, Acad. G. Bonchev Str., Build. 9, 1113 Sofia, Bulgaria; 9 Department of Microbiology, Technological Faculty, University of Food Technologies, 4002 Plovdiv, Bulgaria; tumbarski@abv.bg; 10 Department of Analytical Chemistry and Computer Chemistry, University of Plovdiv, 4000 Plovdiv, Bulgaria

**Keywords:** anthranilic acid, hybrid molecules, in silico, DFT, antiproliferative, antimicrobial, spasmolytic, MEP, molecular docking simulation, anti-inflammatory activity

## Abstract

The present article focuses on the synthesis and biological evaluation of a novel anthranilic acid hybrid and its diamides as antispasmodics. Methods: Due to the predicted in silico methods spasmolytic activity, we synthesized a hybrid molecule of anthranilic acid and 2-(3-chlorophenyl)ethylamine. The obtained hybrid was then applied in acylation with different acyl chlorides. Using in silico analysis, pharmacodynamic profiles of the compounds were predicted. A thorough biological evaluation of the compounds was conducted assessing their in vitro antimicrobial, cytotoxic, anti-inflammatory activity, and ex vivo spasmolytic activity. Density functional theory (DFT) calculation, including geometry optimization, molecular electrostatic potential (MEP) surface, and HOMO-LUMO analysis for the synthesized compounds was conducted using the B3LYP/6–311G(d,p) method to explore the electronic behavior, reactive regions, and stability and chemical reactivity of the compounds. Furthermore, molecular docking simulation along with viscosity measurement indicated that the newly synthesized compounds interact with DNA via groove binding mode. The obtained results from all the experiments demonstrate that the hybrid molecule and its diamides inherit spasmolytic, antimicrobial, and anti-inflammatory capabilities, making them excellent candidates for future medications.

## 1. Introduction

**Figure 1:**
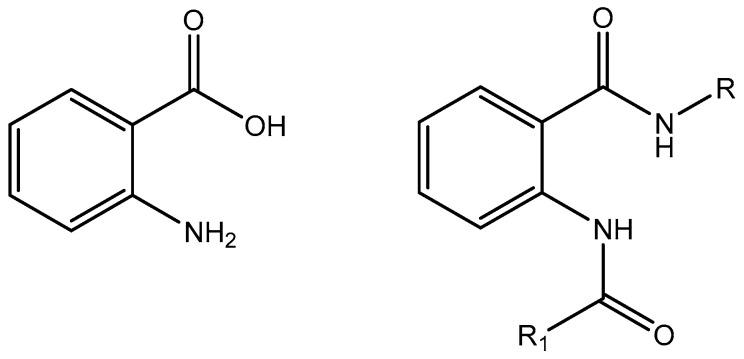
Structure of anthranilic acid and its diamide.

Anthranilic acid and its analogs (Figure 1) are significant components of different bioactive compounds and medications with a variety of biological activities [1,2,3,4]. Functional groups including carboxylic, amino, amide, or ester can interact with their biological targets [5,6,7]. To find the best pharmacophores, these functional groups are used as locations for molecular customizing and structure–activity relationship analysis based on anthranilic acid libraries.

Anthranilic acid and its analogs have an impressive biological profile; this structural nucleus has been thoroughly studied for the development of pharmaceuticals aimed at modulating the biochemical and metabolic pathways contributing to the pathogenesis of various diseases [8,9,10]. In addition to their widespread usage as anti-inflammatory medications, the amides of anthranilic acid are essential in the treatment of many metabolic problems. They also have antiviral, insecticidal, and antibacterial properties. Diamides, on the other hand, have applications as P-glycoprotein inhibitors to manage drug resistance in cancer cells. Additionally, these derivatives function as aldo-keto reductase inhibitors, hedgehog signaling system inhibitors, and apoptosis inducers. The anthranilic acid diamides could be cholecystokinin receptor antagonists [11]. The pancreatic enzyme secretion, gut motility, gall bladder contraction, and gastric emptying are all affected by the hormonal effects of the neurotransmitter cholecystokinin in the gastrointestinal (GI) tract [12,13]. 

**Figure 2:**
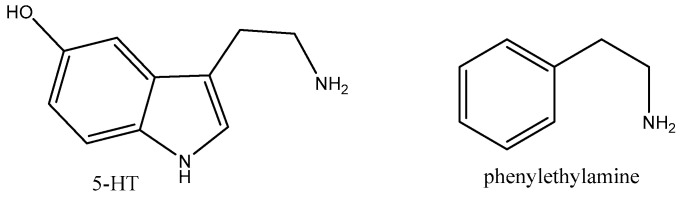
Chemical structures of 5HT and phenylethylamine.

The phenethylamine class, on the other hand, has received the most interest as a 5-hydroxytryptamine (5-HT, serotonin) subtype-selective agonist (Figure 2). Phenethylamines, tryptamines, and ergolines are the three categories into which the 5-HT2A receptor agonist structures often fall [14], but the most attention has been given to the phenylethylamine class. 

The neurotransmitter serotonin plays key roles in mood, libido, aggression, anxiety, cognition, sleep, appetite, and pain in addition to regulating peripheral activities in the circulatory, gastrointestinal, endocrine, and respiratory systems [15,16,17,18]. These activities include appetite, pain, sleep, and appetite regulation.

Studying the intrinsic biological activity of substances in vivo and ex vivo is possible using isolated tissues that are still functionally active. Ex vivo testing is frequently used to confirm the potential biological effect of newly synthesized experimental compounds and approved pharmaceuticals [19]. Because smooth muscle (SM) contraction can still create active tension when separated from the body, we were drawn to include them in the study design we chose [20,21] as a platform for ex vivo contractility evaluation. A number of internal organs’ primary structural components include SM tissue. It is a complex superposition of mechanical activity (tone, frequency, and amplitude of spontaneous or induced muscle contractions) and bioelectrical activity (slow wave with its characteristic frequency and amplitude) that is connected to the motor activity of the stomach and can be measured isometrically in isolated tissues [22,23,24,25]. Given the interest in the field of drug discovery, examining the biological activity of recently synthesized compounds is an important scientific topic. Various substances, including 1,2,3,4-tetrahydroisoquinolines [26], isoquinoline precursors [20], and eucalyptol [27], among others, have been the subject of our prior publications on the synthesis and SM activity.

Finding new therapeutic strategies, manipulating established pharmacological targets, or validating novel agents with a different mechanism of action remain the top priority. Based on the pharmacological activities of anthranilic acid derivatives and phenylethylamines, as well as our previous experiments with 2-phenylethylamines [20,21,28], we considered the synthesis of a new hybrid molecule of anthranilic acid with 2-(3-chlorophenyl)ethylamine and its amides as antispasmodics. Density functional theory (DFT) calculation containing geometry optimization, molecular electrostatic potential (MEP) map, and highest occupied molecular orbital-lowest unoccupied molecular orbital (HOMO-LUMO) energy level, as well as a quantum chemical descriptor, were conducted to determine electronic behavior, reactive region, and stability and chemical reactivity of the compounds. Molecular docking simulation of the title compounds and DNA/albumin were applied to estimate the binding affinities in these systems. Based on these structure-based predictions, the ex vivo contractile activity (CA) of the compounds was established. A thorough biological evaluation of the compounds was conducted assessing their in vitro antimicrobial, cytotoxic, and anti-inflammatory activity compared to mebeverine as a known antispasmodic. 

## 2. Results and Discussion

Finding the optimal targets among the abundance of unique potential candidates is one of the major issues of the post-genome age. In pharmaceutical research and development, choosing “the right” biological target could be the most crucial choice made. This is true for both biotherapeutics and small molecules. All the numerous steps between the moment of target selection and the initiation of proof of human clinical trials generally follow a consistent, well-defined path. Screening and hit identification are followed by rounds of optimization based on pharmacological and toxicological testing, which is eventually succeeded by pharmaceutical development and production. 

### 2.1. In Silico Predictions and Synthesis

There are many factors that limit the systematic use of experiments, an essential step for novel drug discovery. To name a few such factors, we could list the abundance of recently produced experimental molecules, the quantitative restrictions of tissue samples, as well as the ethical requirement to restrict animal testing. In this context, it is reasonable to consider that biological investigations could be supplemented and even completely substituted by in silico computer models are both a good supplement to and a viable substitute for biological investigations [29]. For this purpose, we decided to include a number of software products in our current research on novel hybrid molecules as potential drug candidates. To be successfully used as a drug, a molecule must reach its pharmacological target within the body, reach an appropriate concentration at the site of action, and remain in a bioactive form long enough. Many promising biologically active substances fail as medicines due to their poor pharmacokinetics and bioavailability. The absorption, distribution, metabolism, and excretion (ADME) properties of the target compounds in the presented article were estimated in silico using free SwissADME web tools [29]. Many substances fail as medicines due to their poor pharmacokinetics and bioavailability. In our calculations on the expected biological effects, the PASS Online Program (Prediction of Activity Spectra for Substances) was used [30,31,32]. It predicted the potential spasmolytic activity of the target compounds. Based on the in silico predictions, we worked on a synthetic route to synthesize a new hybrid molecule of anthranilic acid with 2-(3-chlorophenyl)ethylamine.

**Scheme 1:**
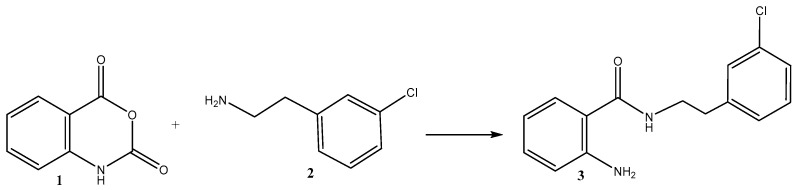
Synthesis of the starting hybrid 2-amino-N-(3-chlorophenethyl)benzamide **3**.

Our synthetic strategy was based on the ring opening of isatoic anhydride **1** with 2-(3-chlorophenyl)ethylamine **2** to form the hybrid molecule **3** with high purity and yield (99%), according to the following Scheme 1. 

The synthesis of a new molecule, containing a phenyl substituent with a chlorine atom attached to a 2-phenylethylamine or substituted 2-phenylethylamines, is extremely interesting in view of what properties the newly obtained molecule would inherit from both fragments. Chloramphenicol, chloroquine, and chlorambucil are just a few examples of medications containing a chlorine atom. These medications are utilized for their respective antibacterial, antibiotic, anticancer, and antimalarial qualities [25].

The structure of the resulting compound was validated using spectral data. In ^1^H-NMR spectra and ^13^C-NMR spectra (Supplementary Materials Figures S1–S4), all the signals were fully consistent with the corresponding molecule. In the ^1^H-NMR broad singlets at 5.49 ppm for the NH_2_ group and 6.65 ppm for NH from the amide group in **3** appeared, as well as a triplet at 2.91 and a quartet at 3.67 ppm for the CH_2_ groups and signals for the aromatic ring of isatoic anhydride and 2-(3-chlorophenyl)ethylamine in the region 6.64–7.29 ppm. In ^13^C-NMR of **3**, the signal for the C=O group at 169.4 ppm appeared, as well as signals for the aromatic ring and CH_2_ groups at 40.6 and 35.5 ppm. FT-IR spectrum also showed bands for NH, NH_2_ groups, amide group, and meta-substituted benzene ring (meta-Ph-Cl).

The target hybrid molecule **3** and its new diamides were successfully synthesized in order to study their biological activity. Due to the prominence of the amide group in biological systems and its crucial function in medicinal chemistry, where amide synthesis is one of the most frequent chemical reactions, amides play a significant role in biological systems, making their synthesis exceedingly vital. The amide functional group’s significance in molecular sciences cannot be overstated. The amide-forming processes, which are the most often used in the pharmaceutical industry [33], are vital in generating the backbone of peptides, proteins, and other macromolecules [34]. An amide bond is present in many therapeutic candidates and about a quarter of all commercially available medications [35]. Another important factor to take into account in the development of new drugs is amide interactions with biological targets [36]. 

**Scheme 2:**
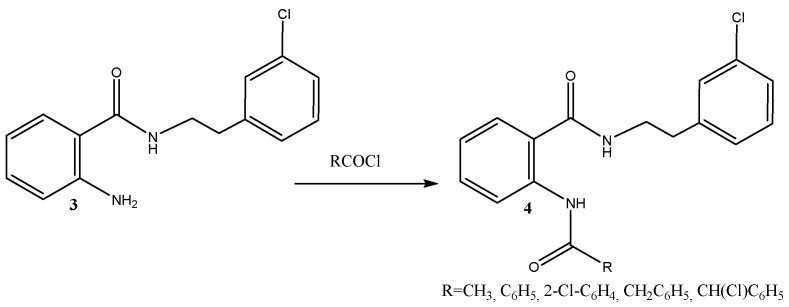
Synthesis of the derived diamides **4a**–**e**.

We developed a synthetic method to use several acyl chlorides having electron-donating and electron-withdrawing groups as starting materials based on the in silico results (Scheme 2).

Reaction with acyl chlorides worked well and efficiently produced the desired diamides **4a**–**e** in 79–83% yield (Table 1).

The resultant compounds are characterized by their melting point, IR, ^1^H, ^13^C-NMR, and HRESIMS spectra. Spectral data confirmed the structure of all the obtained compounds (Supplementary Materials Figures S5–S24). In ^1^H-NMR a broad singlet in the region 10.99–12.1 ppm for the NH from the new amide group in **4a**–**e** appeared, as well as a signal for the new carbonyl group of the second C=O in ^13^C-NMR. 

### 2.2. In Silico Analysis

In drug discovery, virtual screening is a commonly used technique. Docking can be used to thoroughly examine the active site and find undiscovered binding pockets or interaction sites. By contrasting the interactions of ligand molecules when bound inside the protein with those of active drugs, molecular modeling has shown to be a potent method for designing new drug candidates [37]. In our calculations, the computer program SwissADME was used to identify and discard any compounds violating the rules to qualify as a drug. To anticipate mutagenicity, irritation, and reproductive damage, the computer programs OSIRIS20 and ProToxII were utilized. These programmers give overall drug score values to consider the compound as a drug. The biological activities of each compound are predicted using the computer program PASS. It offers a standard for excluding potentially dangerous molecules when choosing compounds for production and experimental testing. It calculates probabilities for the desired pharmacological effect and molecular mechanisms of action. 

Lipophilicity is a key characteristic that plays a major role in assessing bioavailability. According to the in silico screening, predicted by the five proposed methods, the log P values are lower than 5, suggesting good bioavailability (Table 2). The ADME investigation shows that the compounds cross through the blood–brain barrier (BBB) and present high gastrointestinal absorption and could be considered as a drug candidate according to Lipinski’s rule of five [38], an important step in a drug discovery process. The topological polar surface area (TPSA) is a descriptor that provides information about drug transport properties, such as intestinal absorption (TPSA below 140 Å2) and penetration across the BBB [39,40]. The calculated TPSA values of the compounds are in the range of 55.12–58.20 Å2, indicating good intestinal absorption and BBB penetration. The number of rotatable bonds for all the compounds is lower than nine, which corresponds with sufficient oral bioavailability.

Another important property affecting absorption is solubility [41]. The synthesized compounds are moderately soluble in water according to the log S parameter. The bioavailability radar results also confirmed that all the synthesized compounds met the drug-likeness criteria except for the carbon atoms in the sp^3^-hybridization.

Virtual screening shows that the compounds are able to cross the **BBB**, which is a favorable property in the drug discovery process. The compounds have high GI absorption and a probability of 55% for bioavailability. 

A positive feature is that all the compounds are not expected to be substrates of P-glycoprotein. The studied compounds are expected to inhibit cytochromes, which has an adverse effect and is one of the causes of drug interactions leading to drug toxicity [42]. Moreover, all the compounds have good synthetic accessibility scores, which are considerable parameters during the drug discovery processes.

The toxicological properties of compounds were investigated using OSIRIS Property Explorer Software (Available online: http://www.Organic-chemistry.org/prog/peo) [43] and the ProToxII free web tool [44,45]. Both programs assess the mutagenic, tumorigenic, irritant, and reproductive risks of a compound administered as a medicinal product. According to OSIRIS, all the compounds would exhibit low tumorigenic risk or any hazard to the reproductive system. Excluding compound **4e**, which was prognosed to have a high mutagenic risk, the rest of the compounds would have low mutagenicity. Compound **4b** exhibits a medium irritation risk in contrast to the others, which have a low irritation risk. ProToxII on the other hand, predicted all the compounds to be non-toxic and non-mutagenic compounds with LD_50_ ranging between 1000 mg/kg and 2000 mg/kg.

### 2.3. In Vitro Biological Activity

#### 2.3.1. Antimicrobial Activity

Aggregated bacteria that are attached to surfaces and embedded in a self-made matrix of extracellular polymeric materials are known as biofilms. Persister cells within a biofilm are shielded from the immune system by the local environment [46]. Any surface, such as hospital water distribution systems or medical implants, can develop these bacterial biofilms [47]. Those bacteria found in biofilms can be very challenging to get rid of and pose therapeutic issues. One of the most pervasive problems in the world today is antibiotic resistance, and many potent medications have failed to control illnesses. Resistance to antifungal drugs, in the opportunistic yeast *Candida albicans* has become an increasing problem in human immunodeficiency virus (HIV), as well [37,48].

According to the literature, 2-phenylethylamines exerted modest antimicrobial activity against Staphylococcus aureus and *Bacillus subtilis* [49], strong activity against Gram-positive *Bacillus subtilis*, *Mycobacterium smegmatis*, *Listeria monocytogenes*, and *Staphylococcus aureus* and moderate activity against *Candida albicans* [50], as well as against *Listeria monocytogenes* [51].

In our experiments, six Gram-positive bacteria, six Gram-negative bacteria, two yeasts, and six fungi were used. The hybrid molecule and its diamides were tested in vitro for their antimicrobial activity against human pathogenic bacteria and economically relevant phytopathogenic fungi. The inhibition zones of bacterial and fungal growth caused by the novel compounds are outlined in Table 3. The obtained results were compared to a known antispasmodic mebeverine and its precursors [21]. The methanol used as a solvent for the samples did not show any antimicrobial effect. 

We observed that the hybrid molecule **3** derivative exerted very good antimicrobial activity and its diamides have modest activity against Gram-positive *Micrococcus luteus*, Gram-negative bacteria including the most pathogenic *Klebsiella* sp., *Pseudomonas aeruginosa*, as well as against yeasts *Saccharomyces cerevisiae* and *Candida Albicans*. This trend led us to the conclusion that antibacterial properties are distinctive to hybrid molecule **3**, whereas diamides **4a**–**e** show modest antimicrobial activity. The newly synthesized compounds showed better antimicrobial activity than previously described mebeverine precursors [21]. A known antispasmodic mebeverine did not show any antimicrobial activity in the same concentration range. This gave us an opportunity to further investigate the in vitro, ex vivo, and in vivo biological activities of the synthesized compounds.

#### 2.3.2. Cytotoxicity

Based on the PASS online and the ProToxII in silico calculations, the evaluation of the cytotoxic activity of the compounds was the other object of our investigation. A series of MTT experiments were conducted to evaluate the in vitro effects of the tested compounds on cell growth in both normal (murine fibroblast CCL-1 cells) and malignant human cell lines of leukemic (K-562, LAMA-84) and epithelial (T-24 bladder carcinoma cells) origin. Results of the cell viability assays are presented in Table 4. 

According to our study, cell proliferation was poorly or not at all affected by the hybrid compound **3** and its diamide derivatives **4a**–**4c**. Most of the estimated IC_50_ values lie near and above 100 µM, indicating minimal inhibitory activity, especially on the adherently growing T-24 and CCL-1 cell cultures. An isolated slightly higher susceptibility to two of the analogs in the series (**4b** and **4c**) was observed in the leukemic cells LAMA-84 and K-562 with IC_50_ estimates ranging between 30 and 60 µM. However, such regularity by cell type is foreseeable, as suspension-grown cells are generally characterized by greater chemosensitivity due to the larger membrane cell surface and easier access of drugs to the cell. An overall non-toxicity was also established in the normal murine fibroblast cell line CCL-1, where four out of the six compounds yielded undefinable half-inhibitory concentrations. The obtained results showed that compounds **3**, **4a**, **4d**, and **4e** showed a more favorable cytotoxicity profile, compared to mebeverine, a spasmolytic drug used as a reference, whose half-inhibitory concentrations fall under the hundred-micromolar range [21]. 

#### 2.3.3. Inhibition of Albumin Denaturation

**Figure 3:**
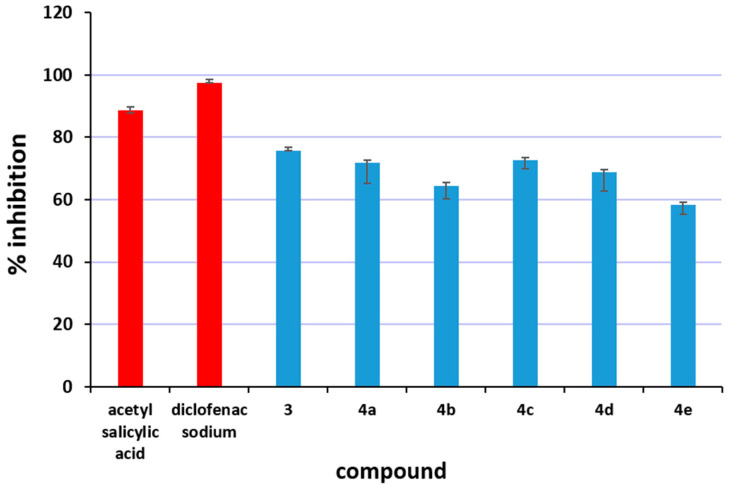
Percent inhibition of albumin denaturation–compounds **3**, **4a**–**e** (blue), diclofenac and acetylsalicylic acid (red) are used as standards.

According to modern understanding, inflammation is a healthy process that happens in reaction to a disruption or illness. The ability to stop inflammation or swelling is an anti-inflammatory property of medication or therapy. Anti-inflammatory drugs reduce pain by lowering inflammation. There are two classes of pharmaceuticals that are widely used to treat inflammation: steroidal anti-inflammatory drugs and non-steroidal anti-inflammatory drugs. Non-steroidal ones have a number of unfavorable side effects, especially GI disorders that can lead to gastric ulcers [52]. This work aimed to create novel antispasmodics that prevent albumin denaturation. In vitro, analysis of anti-inflammatory activity was assessed as inhibition of albumin denaturation estimating the degree of denaturation resistance of the albumin molecule. Anti-denaturation of the human albumin method was used to evaluate the anti-inflammatory properties of compounds **3** and **4a**–**e** (Figure 3). All the compounds protected the human albumin against heat-induced denaturation showing anti-inflammatory activity better than diclofenac and acetylsalicylic acid. 

The IC_50_ values for all the compounds and inhibition of albumin denaturation are presented in Table 5. The obtained data showed a concentration-dependent inhibition of protein (albumin) denaturation by hybrid molecule **3** and its diamides **4a**–**e** with IC_50_ from 650 to 830 μg/mL.

The presented results point out that all the compounds protected the human albumin against heat-induced denaturation demonstrating better anti-inflammatory activity compared to two well-established non-steroidal anti-inflammatory drugs diclofenac sodium and acetylsalicylic acid. We found that the highest albumin protection activity is expressed by compounds **3** and **4a**, followed by **4c** and **4d**. Compound **4e** appears to be less active, as its IC_50_ was found to be the highest.

We assumed that the higher activity of the newly synthesized compounds is attributed to the presence of a 2-phenylethylamine group, as well as possible hydrogen bonding with the amide group, oxygen, or chlorine to the albumin macromolecule. To explain better the anti-inflammatory activity results, molecular docking simulations were established.

### 2.4. DFT Perspective

**Figure 4:**
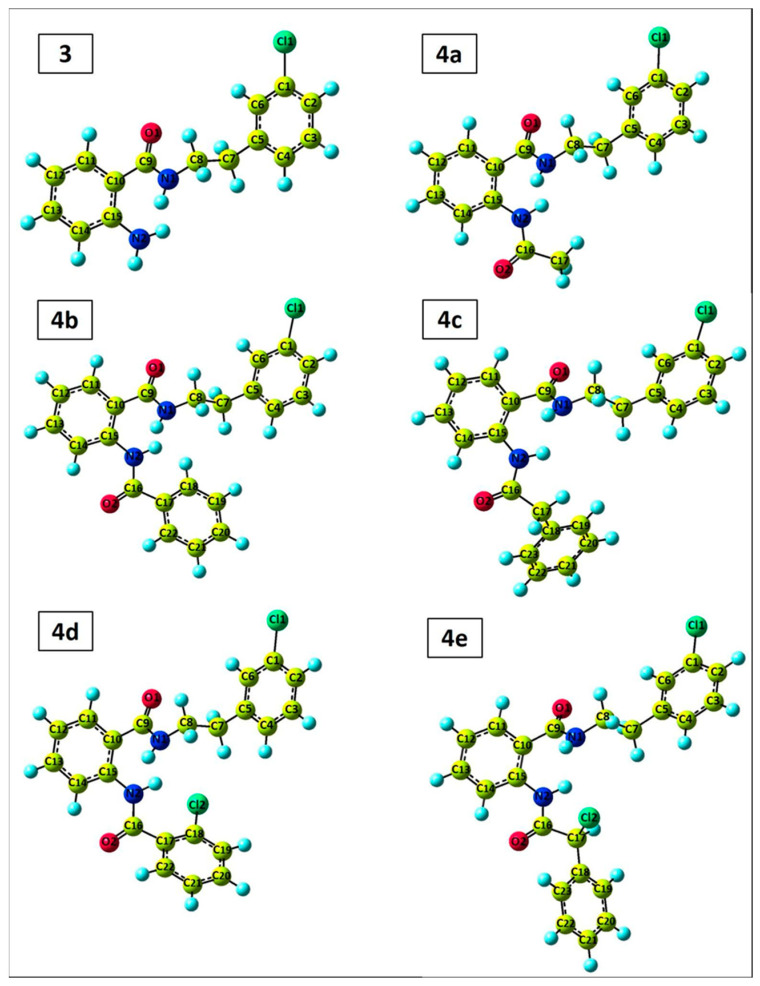
The Optimized structures of **3** and **4a**–**e** compounds along with atom numbering obtained from DFT.

The structure of **3** and **4a**–**e** was optimized using DFT/B3LYP/6–311G(d,p) level of calculation. Figure 4 demonstrates the optimized geometry of these compounds with the lowest energy level. The calculated electronic energy for **3** and **4a**–**e** is found to be −1225.82, −1378.52, −1570.30, −1609.61, −2029.89, and −2069.22 Hartree, respectively. These results indicate that the **4e** compound has more negative electronic energy than other compounds.

**Figure 5:**
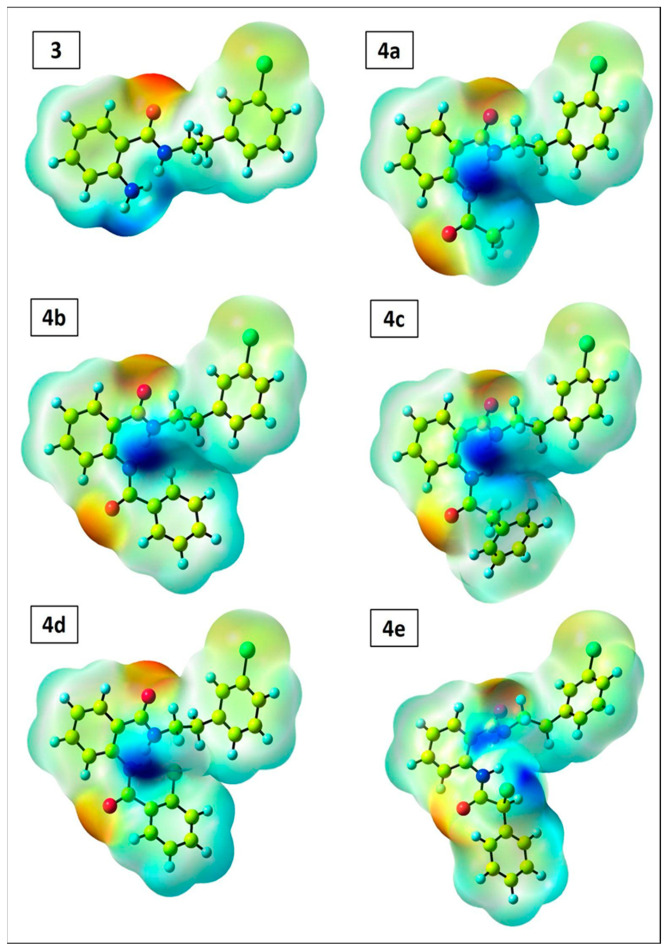
The MEP surface of **3** and **4a**–**e** compounds using DFT/B3LYP/6–311G(d,p) level of theory.

The binding rate of a small compound to a macromolecule such as DNA is strongly influenced by the partial charges on the compound. The molecular electrostatic potential (MEP) map can be utilized for a better representation of the partial charge on the molecule. Therefore, with the help of the MEP map, it is possible to predict the active regions of the compounds for interaction with macromolecules. To supply more details into the nucleophilic and electrophilic regions of **3** and **4a**–**e** compounds, the MEP surface was obtained via the DFT/B3LYP/6–311G(d,p) method as illustrated in Figure 5. MEP diagram is classified by various colors, from red to blue, and colors between the two. The red zone in the diagram denotes the electron-rich area, which is called the nucleophilic site (negative region), while the blue one illustrates the electrophilic site (positive region). The green area represents the neutral region, as we previously described [53]. The MEP diagram of compound **3** indicates that the blue color is located around the N1H and N2H_2_ groups, which is the proper group for the electrophilic attack on the negative sites of the macromolecules. On the other hand, the red color is concentrated on the C9=O1 group, suggesting this area is related to the nucleophilic site and can create hydrogen bonding with the macromolecule. Due to the green color around the C1–Cl1 group, this area is considered a neutral site. According to the MEP map of **4a**–**e** compounds, N1H and N2H groups are electrophilic sites, and C9=O1 and C16=O2 are nucleophilic zones.

**Figure 6:**
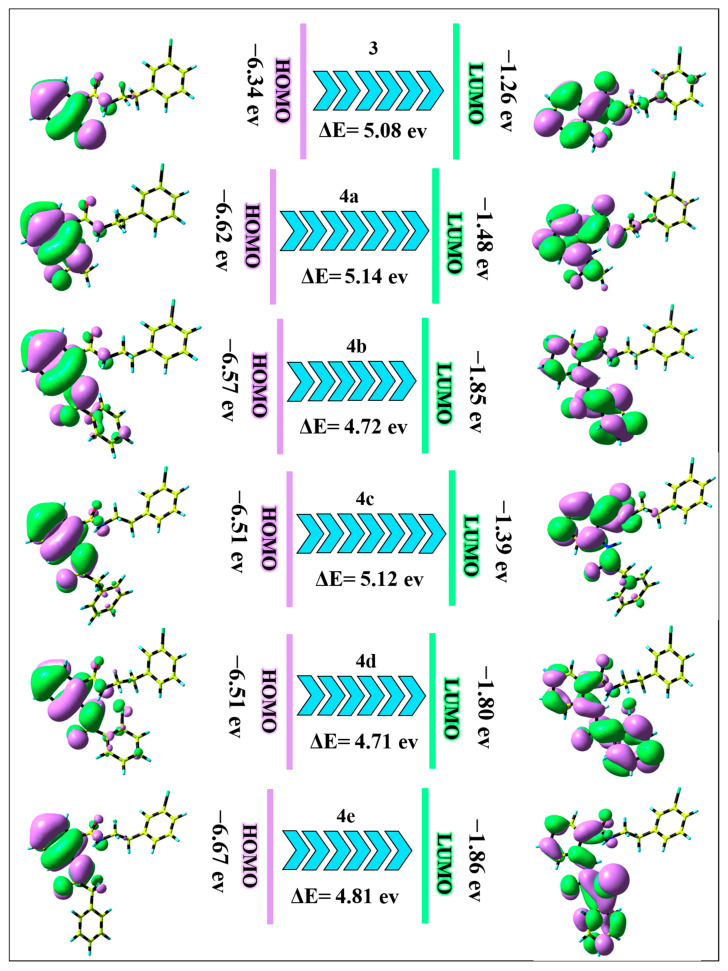
The frontier molecular orbitals (HOMO-LUMO) of **3** and **4a**–**e** compounds.

The HOMO-LUMO energy level can be used as a vital parameter for explaining the electronic behavior of the synthesized compounds. The HOMO and LUMO are the fundamental orbitals responsible for the chemical reactivity and stability of a molecule [54]. Figure 6 summarizes the estimated HOMO-LUMO energy levels along with its energy gap (ΔE) for **3** and **4a**–**e** compounds. In molecule **3**, the electronic cloud of HOMO and LUMO orbitals is concentrated on the anthranilic acid part. In **4a**–**e** molecules, the HOMO orbital is situated on the anthranilic acid part, and the LUMO orbital is localized on the anthranilic acid and its related amide group I. The values of HOMO-LUMO energy level for the newly synthesized compounds are desirable evidence for charge transfer of HOMO → LUMO. Generally, the compound including a small value of ΔE is correlated with low stability and high chemical reactivity. The energy of HOMO for **3**, **4a**–**e** compounds consist of −6.34, −6.62, −6.57, −6.51, −6.51, and −6.67 eV, respectively due to the π-orbital in the anthranilic acid part, while the energy of LUMO for these compounds is due to the π*-orbital consist of −1.26, −1.48, −1.85, −1.39, −1.80 and −1.86 eV, respectively [55]. The small value of ΔE was observed for all synthesized compounds, indicating the probability of electron transition from π orbital to π* (HOMO → LUMO). Furthermore, the values of the energy gap are in the form of **4a** (5.14) > **4c** (5.12) > **3** (5.08) > **4e** (4.81) > **4b** (4.72) > **4d** (4.71). So, the trend of the chemical reactivity and stability of these compounds is **4d** > **4b** > **4e** > **3** > **4c** > **4a** and **4a** > **4c** > **3** > **4e** > **4b** > **4d**, respectively. 

The additional quantum chemical interpretation that illustrates the chemical reactivity of the compound like absolute hardness (η = (E_LUMO_ – E_HOMO_)/2), absolute softness (σ = 1/η), absolute electronegativity (χ = −(E_HOMO_ + E_LUMO_)/2), chemical potential (P_i_ = −χ), additional electronic charge (ΔN = −P_i_/η), and global electrophilicity (ω = P_i_^2^/2η), were also calculated and summarized in Table 6. The P_i_ parameter for all synthesized compounds is found to be negative, revealing that the structure of these compounds does not disintegrate into primal elements. The global electrophilicity index illustrates the capability of electron acceptors to obtain additional electronic charge from the system. The values of ω for **4a**–**e** compounds (3.19, 3.75, 3.05, 3.66, and 3.78, respectively) are higher than compound **3**, demonstrating these compounds can create more binding mode with DNA and albumin. The ΔN parameter describes the number of electron transfers and demonstrates the ability of a compound to gain electrons from another molecule. As a result, **4a**–**e** compounds possess the best and highest value of the number of electron transfers than compound **3**. Furthermore, the thermodynamic parameters including thermal energy (E_T_), heat capacity (C_V_, cal/molK), and entropy (S, cal/molK) were obtained for the present compounds according to the DFT/B3LYP/6–311G(d,p) method and the results are listed in Table 6. These parameters were determined in the condition of T = 298.07 K and P = 1.00 atm.

### 2.5. Molecular Docking Simulation

#### 2.5.1. DNA Simulation

**Figure 7:**
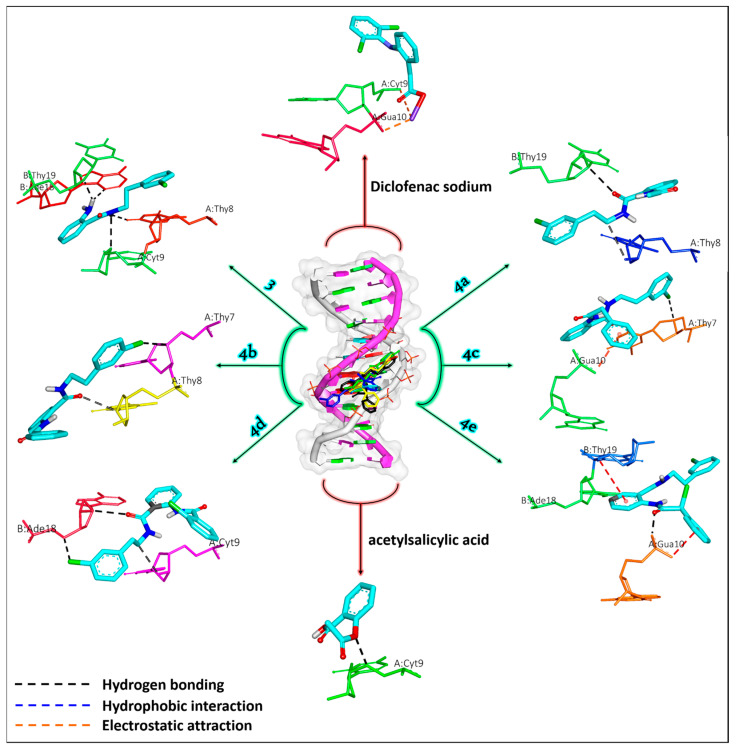
The molecular docking representation of the newly synthesized compounds **3** and **4a**–**e,** as well as diclofenac sodium and acetylsalicylic acid as positive controls with DNA.

Molecular docking is a unique perspective to exhibit the interactions of small compounds with macromolecules. Due to the promising in vitro biological activity of the synthesized compounds, the interactions of these compounds with DNA and albumin were investigated utilizing in silico molecular docking simulation. Furthermore, docking simulations of diclofenac sodium and acetylsalicylic acid as positive controls with DNA were conducted. Based on docking simulation, diclofenac sodium, acetylsalicylic acid, **3** and **4a**–**e** compounds interact with DNA duplex with binding free energy (ΔG) of −4.75, −2.43, −5.63, −6.24, −6.15, −6.35, −6.54, and −6.49 kcal/mol, respectively. The obtained value of ΔG for these compounds shows that these compounds are biologically more active than diclofenac sodium and acetylsalicylic acid and can form proper interaction with DNA in comparison with diclofenac sodium and acetylsalicylic acid. Of the 10 poses generated for synthesized compounds, the one with the best binding mode (with more negative ΔG) was chosen for docking analysis. Docking analysis indicates that all compounds fit into the minor groove of DNA (Figure 7). Compound **3** forms four hydrogen bonds with Ade18 (distance: 2.42 Å), Thy19 (distance: 2.17 Å), Cyt9 (distance: 3.01 Å), and Thy8 (distance: 2.40 Å) base pairs of DNAs. Compound **4a** creates two hydrogen bonds with Thy19 (3.13 Å) and Thy8 (3.30 Å) base pairs. Compound **4b** interacts with Thy7 (3.11 Å) and Thy8 (3.15 Å) base pairs via hydrogen bonding. Compound **4c** forms one hydrogen bond with the Thy7 (3.25 Å) base pair and one electrostatic interaction with the Gua10 (3.22 Å) base pair of DNAs. Compound **4d** binds to Ade18 (2.70 and 3.03 Å) with two hydrogen bonds and Cyt9 (2.96 Å) with one hydrogen bond. Compound **4e** interacts with Gua10 via one electrostatic attraction (3.28 Å) and one hydrogen bond (2.10 Å) and Thy19 via one electrostatic attraction (3.48 Å). Diclofenac sodium binds with Gua10 (2.69 Å) and Cyt9 (3.79 Å) via electrostatic attraction. Acetylsalicylic acid creates one hydrogen bond with Cyt9 (2.78 Å). 

As a result, docking simulations demonstrate that hydrogen bonding plays a crucial role in the binding process of the newly synthesized compounds with DNA. In short, DNA is the major target of an antitumor agent. So, the interaction of antitumor agents with DNA will complementarily help pharmacological understanding of the use of these agents. The stronger interaction of the compounds with DNA may lead to higher conformational change and prevent replication and transcription [56]. This process prevents the replication of damaged DNA. Thus, it can be concluded that these compounds may have antitumor activities due to their strong interaction with DNA. This gives us an opportunity to further investigate in vivo biological activities of the synthesized compounds.

#### 2.5.2. Albumin Simulation

**Figure 8:**
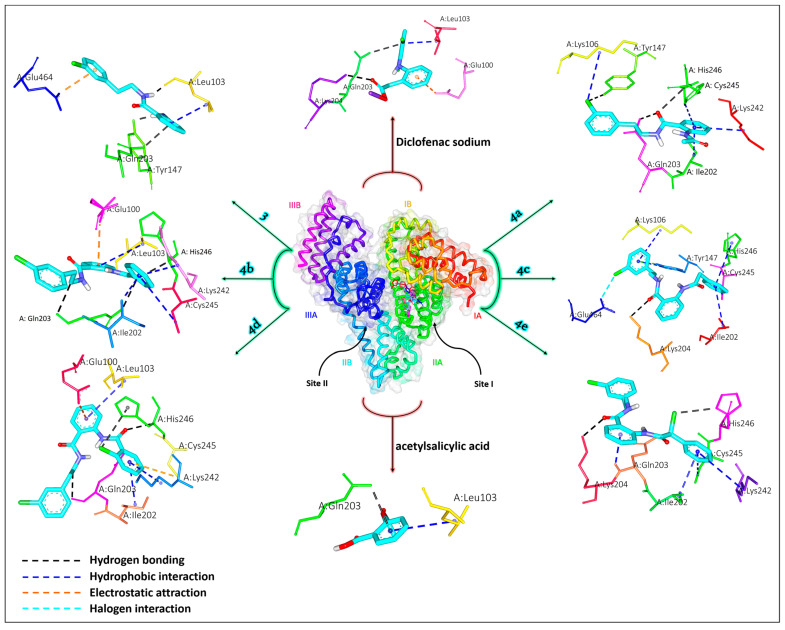
The molecular docking representation of the newly synthesized compounds **3** and **4a**–**e** as well as diclofenac sodium and acetylsalicylic acid as positive controls with albumin.

Serum albumin is the main soluble protein present in the blood plasma that carries various compounds such as drugs in the blood circulation system. The albumin’s structure contains three important domains, i.e., I, II, and III, and each domain is made up of two subdomains (A and B). Also, the structure of this protein has two specific sites for drug binding (drug binding sites I and II). Molecular docking simulations of the synthesized compounds with albumin were performed in order to determine the nature of the interaction in these systems and binding affinities, as well as to explain the anti-inflammatory activities of the newly synthesized compounds. Docking simulations were applied for two drug binding sites and the results indicated that the ΔG value for the drug binding site I is more negative than drug binding site II. So, the drug binding site I was selected for docking analysis. Docking reveals that the title compounds interact with albumin via drug binding site I (Figure 8). The estimated value of ΔG for diclofenac sodium, acetylsalicylic acid, **3** and **4a**–**e** compounds are found as −4.05, −2.76, −5.74, −5.57, −4.75, −5.31, −5.14, and −4.85 kcal/mol, respectively. The **3**, **4a**, **4c**, and **4d** compounds possess higher binding affinities than the **4b, 4e**, acetylsalicylic acid, and diclofenac sodium, which align with the in vitro findings. Compound **3** interacts with albumin via three hydrogen bonds with Gln203 (distance: 3.07 Å), Tyr147 (distance: 1.85 Å), and Leu103 (distance: 1.92 Å) residues, one hydrophobic interaction (Pi-Alkyl) with Leu103 (3.47 Å), and one electrostatic attraction (Pi-Anion) with Glu464 (3.49 Å) residue. Compound **4a** creates three hydrogen bonds with Tyr147 (2.98 Å), His246 (3.19 Å), and Gln203 (2.70 Å) residues, and four hydrophobic interactions with Lys106 (3.93 Å), Cys245 (3.98 Å), Lys242 (3.76 Å), and Ile202 (3.34 Å) residues of albumin. Compound **4b** binds with albumin via three hydrogen bonds with Gln203 (2.99 Å), His246 (3.11 Å), and Gln203 (3.00 Å), four hydrophobic interactions with Leu103 (3.70 Å), Ile202 (3.45 Å), Lys242 (3.59 Å), and Cys245 (3.98 Å), and one electrostatic attraction with Glu100 (3.25 Å) residue. Compound **4c** interacts with albumin by one hydrogen bond with Lys204 (2.98 Å), five hydrophobic interactions with Ile202 (3.56 Å), Cys245 (3.32 Å), His246 (3.67 Å), Tyr147 (2.89 Å), and Lys106 (3.58 Å), and one halogen bond with Glu464 (3.07 Å) residue. Compound **4d** forms three hydrogen bonds with His246 (2.95 Å), Gln203 (3.01 Å), and His246 (2.97 Å), three hydrophobic interactions with Leu103 (3.34 Å), Lys242 (3.59 Å), and Ile202 (3.39 Å), and two electrostatic attractions with Cys245 (3.89 Å) and Glu100 (3.46 Å) residues of albumin. Compound **4e** binds albumin by two hydrogen bonds with Lys204 (2.97 Å) and His246 (3.02 Å) residues, and four hydrophobic attractions with Lys204 (2.85 Å), Ile202 (3.28 Å), Cys245 (3.36 Å), and Lys242 (3.43 Å) amino acid residues. Docking simulation demonstrates that hydrogen bonding and hydrophobic interaction play a vital role in the nature of the binding between synthesized compounds and albumin. Diclofenac sodium interacts with Lys204 (2.85Å) and Gln203 (3.17 Å) via hydrogen bonding, Leu103 (3.27 Å) via hydrophobic interaction, and Glu100 (2.34 Å) residue by electrostatic attraction. Acetylsalicylic acid forms one hydrogen bond with Gln203 (3.16 Å) and one hydrophobic interaction with Leu103 (3.37 Å).

The obtained data confirmed the experimental results from in vitro anti-inflammatory activity. The results showed that the most active were hybrid **3** and its diamide **4a**. This data can be explained by the higher amount of hydrogen bonding provided by the structures to stabilize the albumin macromolecule. These interactions are responsible for preventing albumin denaturation during inflammatory processes [57,58,59,60]. Furthermore, hydrogen bonds are generated with oxygen or chlorine with polar amino acid residues. These results completely confirmed the in silico studies for anti-inflammatory effects. The aforementioned demonstrates that the hybrid molecule and its diamides inherit anti-inflammatory capabilities, better than diclofenac, making them excellent candidates for future medications. 

### 2.6. Viscosity Measurement

**Figure 9:**
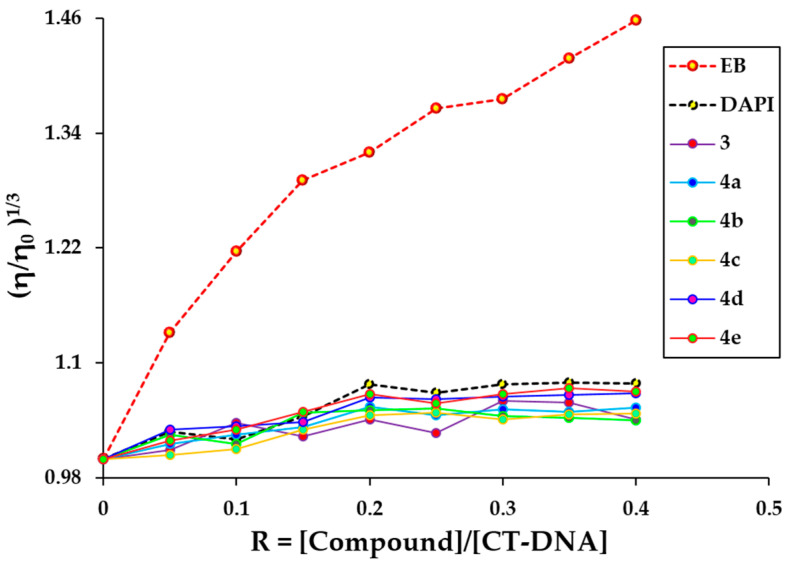
The effect of various concentrations of EB, DAPI, **3**, and **4a**–**e** compounds on the viscosity of CT-DNA.

Viscosity measurement was performed to investigate the interaction type of the synthesized compounds (**3**, **4a**–**e**) with DNA. During the interaction of groove binders including DAPI with DNA, these molecules may slightly increase the viscosity of DNA or may not change much [61]. In contrast, during the interaction of classical intercalators such as EB (ethidium bromide) with DNA, these compounds are situated between DNA base pairs and lead to strong interaction and an increase in DNA viscosity [62]. The effect of rising concentration of EB, DAPI, **3**, and **4a**–**e** compounds on the relative viscosity of DNA is illustrated in Figure 9. 

The viscosity of DNA remains approximately constant or increases slightly with the addition of increasing concentrations of **3**, **4a**–**e**, which is a distinctive feature of groove binding mode. On the other hand, the behavior of **3**, and **4a**–**e** compounds is similar to DAPI in changing the viscosity of DNA, indicating these new compounds interact with DNA via groove binding. This finding supports the obtained result from DNA docking simulation. 

### 2.7. Ex Vivo Smooth Muscle Relaxant Activity

In vitro and ex vivo model systems can serve as useful tools in the fundamental research on novel medications to explore concentration-response relationships that depend on the effects on GI muscle activity. Pharmacologically pre-contracted smooth muscle (SM) preparations are exposed to bioactive substances in the tissue bath models [63]. The effectiveness and potency of contractile agonists can be altered by raising the concentrations of their inhibitors or antagonists after therapy. This allows monitoring of concentration-dependent changes to isometrically registered contraction [64].

**Figure 10:**
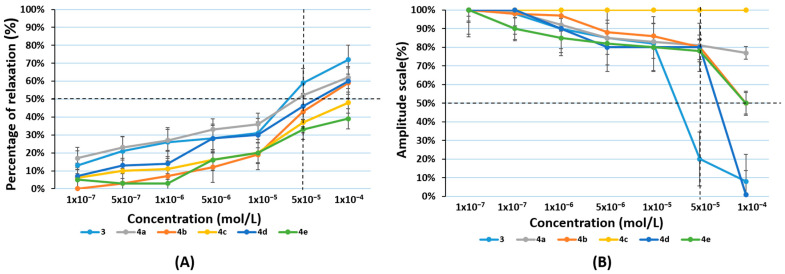
(**A**) Percentage of relaxation induced by compounds **3** and **4a**–**e** in the concentration range from 1 × 10^−7^ to 1 × 10^−4^ mol/L on pre-contracted SM tissues with 1 × 10^−7^ mol/L CCh. Contractile responses induced by CCh were taken as the 100% values, and all subsequent responses were expressed as a percentage of this value. (**B**) Percentage variation in the size of amplitudes of spontaneous CA before and after the application of substances **3** and **4a**–**e**. (n = 5).

The utilization of an SM preparation from the corpus of a rat stomach that had been pharmacologically pre-contracted was the main goal of our work. The excitatory neurotransmitter acetylcholine (ACh) in the enteric nervous system is appropriate for this purpose [65]. It affects the digestive system by increasing the amplitude of digestive contractions and peristalsis in the stomach. We evaluated the viability of the SM using their stimulatory impact. Via muscarinic (M2 and M3) receptors, ACh and its counterpart cholinergic agonist carbachol (CCh, carbamylcholine) cause excitatory motor responses in GI SM cells [66]. Unlike ACh, which breaks down quickly, CCh has a long-lasting response. Therefore, we used CCh to start the induced muscle contraction. The SM tissue exhibits an initial spiking component (change in phase contraction) following this cholinergic stimulation as a result of intracellular Ca^2+^ mobilization. A persistent, prolonged tonic contraction that is brought on by the extracellular inflow of Ca^2+^ into SM cells occurs next [67]. The relaxation effects of the tested compounds **3** and **4a**–**e** were investigated by cumulative addition of the tested substances in concentrations of 1 × 10^−7^, 5 × 10^−7^, 1 × 10^−6^, 5 × 10^−6^, 1 × 10^−5^, 5 × 10^−5^, and 1 × 10^−4^ mol/L at the plateau of the CCh-induced contraction. In order to measure relaxation, the percent decrease in contractile tension from the 1 × 10^−7^ mol/L CCh-induced maximum was used. All subsequent reactions to chemicals were reported as a percentage of the submaximal SM contractile responses elicited by CCh, which were taken as 100% values and expressed as a base value for all subsequent responses (Figure 10A). Results were presented as mean ± SD.

We found that all the tested compounds caused a relaxation effect. These results completely confirmed the character of those groups as compounds with potential spasmolytic activity. Compounds **3** and **4a**–**e** caused inhibition of spontaneous contractions of the rat stomach (with values ranging from 0% to 72%), which was concentration-dependent. We observed equivalent variations in contraction amplitudes when monitoring the other two key elements (frequency and amplitude) of spontaneous CA (Figure 10B). We found no significant changes in contraction frequency for any of the tested compounds. The most pronounced concentration-dependent relaxation effect showed only compounds **3** and **4a**. 

Tonic relaxations of 59% were produced by compound **3** at submaximal concentrations of 5 × 10^−5^ mol/L. The relaxation effects brought by compound **4a** therapy at the same concentrations were 52% (*p* < 0.05), respectively, demonstrating that hybrid molecule **3** provided better relaxation than its diamide **4a**. We found that only the hybrid molecule **3** affects the amplitude of spontaneous contractions by more than 50% at a submaximal concentration of 5 × 10^−5^ mol/L, which allows us to conclude that it is the most effective compound.

## 3. Materials and Methods

### 3.1. Chemicals

All solvents and reagents were purchased from Merck (Merck KGaA, Darmstadt, Germany). Ethidium bromide (EB), DAPI (4′,6-diamidino-2-phenylindole), highly polymerized CT-DNA (type 1), and Tris–HCl buffer were obtained from Aldrich company.

Melting points were determined on a Boetius hot stage apparatus and were uncorrected. All the compounds were characterized by ^1^H NMR, ^13^CNMR, IR, and HRESIMS. The purity of these compounds was determined by TLC using several solvent systems of different polarity. TLC was carried out on precoated 0.2 mm Fluka silica gel 60 plates (Merck KGaA, Darmstadt, Germany), using chloroform:diethyl ether:n-hexane = 6:3:1 as a chromatographic system. Neutral Al_2_O_3_ was used for column chromatographic separation. The products, after evaporation of the solvent, were purified by recrystallization from diethyl ether.

IR spectra were determined on a VERTEX 70 FT-IR spectrometer (Bruker Optics, Ettlingen, Germany). ^1^H NMR and ^13^C NMR spectra were recorded on a Bruker Avance III HD 500 spectrometer (Bruker, Billerica, MA, USA) at 500 MHz (^1^H-NMR) and 125 MHz (^13^C-NMR), respectively. Chemical shifts are given in relative ppm and were referenced to tetramethylsilane (TMS) (δ = 0.00 ppm) as an internal standard; the coupling constants are indicated in Hz. The NMR spectra were recorded at room temperature (ac. 295 K). HRESIMS spectra were acquired in positive mode on Q Exactive Plus (ThermoFisher Scientific, Inc., Bremen, Germany) mass spectrometer, equipped with a heated HESI-II source. Operating conditions for the HESI source used in a positive ionization mode were: +3.5 kV spray voltage, 320 °C capillary and probe heater temperature, sheath gas flow rate 36 a.u., auxiliary gas flow rate 11 a.u., spare gas flow rate 1 a.u. [a.u. refer to arbitrary values set by the Exactive Tune software 2.4] and S-Lens RF level 50.00. Nitrogen was used for sample nebulization and collision gas in the HCD cell. The aliquots of 1 µL of the solutions of the samples (ca. 20 µg mL^−1^) were introduced into the mass spectrometer via LC system Thermo Scientific Dionex Ultimate 3000 RSLC (Thermo Fisher Scientific, Germering, Germany) consisting of 6-channel degasser SRD-3600, high-pressure gradient pump HPG-3400RS, autosampler WPS-3000TRS, and column compartment TCC-3000RS equipped with narrow bore Hypersil GOLD™ C18 (2.1 × 50 mm, 1.9 μm) column. Each chromatographic run was carried out isocratically with a mobile phase consisting of water-acetonitrile-methanol-acetic acid (25:50:25:0.2). The solvent flow rate was 300 μL min^−1^. Full MS—SIM was used as an MS experiment in negative and positive mode, where resolution, automatic gain control (AGC) target, maximum injection time (IT), and mass range were 70,000 (at *m*/*z* 200), 3e6, 100 ms, and *m*/*z* 100–500, respectively. Xcalibur (Thermo Fisher Scientific, Waltham, MA, USA) ver. 4.0 was used for data acquisition and processing.

### 3.2. Synthetic Methods Experimental Protocols and Spectral Data

#### 3.2.1. Synthesis of the Hybrid Molecule 2-Amino-N-(3-chlorophenethyl)benzamide 3

A mixture of isatoic anhydride (1.63 g, 10 mmol) and 2-(3-chlorophenyl)ethylamine (2.10 mL, 15 mmol) in dichloromethane (30 mL) was stirred overnight at rt. The resulting solution was filtered on the neutral Al_2_O_3_ and concentrated. Spectral data confirmed the structure of the hybrid molecule **3** (Supplementary Materials Figures S1–S4).

2-amino-N-(3-chlorophenethyl)benzamide (**3**): ^1^H-NMR: 2.91 (t, *J* = 7.1, 2H, CH_2_), 3.67 (q, J = 5,10, 2H, CH_2_) 5.46 (broad s, 2H, NH_2_), 6.15 (s, 1H, NH), 6.64 (td, J = 7.6, 1H, Ar), 6.68–6.70 (m, 1H, Ar), 7.13–7.15 (m, 1H, Ar), 7.20 (s, 1H, Ar), 7.21 (s, 1H, Ar), 7.25–7.26 (m, 2H, Ar), 7.29 (s, 1H, Ar); ^13^C-NMR: 169.4, 148.7, 141.08, 134.5, 132.35, 129.96, 129.95, 127.05, 126.8, 117.3, 116.08, 116.04, 40.6, 35.5. FT-IR, cm^−1^: 3434 ν(N–H, -NH_2_), 3332 v (-N-H, -NH_2_), ν(-N-H, >NH-amide), 3065 ν(C_sp_^2^-H, -Ph), 3065 ν(C-H, Ph), 2935 ν^as^(C–H, CH_2_), 1645 v (>C=O), secondary amide I, 1536 δ(N-H) + v(C-N), trans-secondary amide II, 1294 ν(C-N), secondary amide III, 1082 δ (meta-Ph-Cl); HRMS Electrospray ionization (ESI) *m*/*z* calcd for [M + H]^+^ C_15_H_17_ClN_2_O^+^ = 275.09457, found 275.09418 (mass error ∆m = −1.41 ppm)

#### 3.2.2. Preparation of Diamides of the Hybrid Molecule **4a**–**e**; Typical Procedure

To a solution of 3 mmol 2-amino-N-(3-chlorophenethyl)benzamide **3**, 3.5 mmol of the corresponding acyl chloride in dichloromethane (10 mL) was added. Then, 3.4 mmol N(C_2_H_5_)_3_ was added in 10 min. In about 30 min the reaction mixture was washed consequently with diluted HCl (1:4), Na_2_CO_3_, and H_2_O, then dried with anhydrous Na_2_SO_4_, filtered on the short column filled with neutral Al_2_O_3,_ and concentrated.

2-acetamido-N-(3-chlorophenethyl)benzamide (**4a**): ^1^H-NMR: 2.19 (s, 3H, COCH_3_), 2.94 (t, J = 5, 2H, CH_2_), 3.68 (q, J = 5, 10, 2H, CH_2_), 6.65 (broad s, 1H, NH), 7.01–7.04 (m, 1H, Ar), 7.13 (d, J = 6.8, 1H, Ar), 7.25–7.28 (m, 3H, Ar), 7.35–7.37 (m, 1H, Ar), 7.43 (t, J = 8.8, 1H, Ar), 8.52 (d, J = 8.3, 1H, Ar), 10.99 (broad s, 1H, NH); ^13^C-NMR: 169.07, 140.7, 139.4, 134.5, 134.4, 132.48, 130.0, 129.9, 128.9, 127.0, 126.9, 126.5, 121.5, 120.4, 40.87, 40.7, 35.24, 35.17, 25.3, 22.8; FT-IR, cm^−1^: 3245 (-N-H, >NH-amide), 3056 ν(C_sp_^2^-H, -Ph), 2976 ν_as_ (C_sp_^3^-H, -CH_3_), 2934 ν^as^(C–H, CH_2_), 1664 ν(>C=O), secondary amide I, 1600 ν(C-C=C, -Phmeta), 1570 δ(N-H) + ν(C-N), trans-secondary amide II, 1527 δ(N-H) + ν(C-N), trans-secondary amide II, 1488 ν(C-C=C, -Phmeta), 1453 ν(C-C=C, -Phortho), 1441 ν(C-C=C, -Phmeta), 1430 ν(C-C=C, -Phortho), 1363 δs(-CH_3_), 1304 v(C-N), secondary amide III, 1285 ν(C-N), secondary amide III, 1082 δ (meta-Ph-Cl); HRMS Electrospray ionization (ESI) *m*/*z* calcd for [M + H]^+^ C_17_H_19_ClN_2_O_2_^+^ = 317.10513, found 317.10469 (mass error ∆m = −1.39 ppm).

2-benzamido-N-(3-chlorophenethyl)benzamide (**4b**): ^1^H-NMR: 2.95 (t, J = 6.8, 2H, CH_2_), 3.71–3.75 (m, 2H, CH_2_), 6.69 (broad s, 1H, NH), 7.02–7.06 (m, 1H, Ar), 7.13 (d, J = 7.3, 1H, Ar), 7.40–7.44 (m, 1H, Ar), 7.48–7.59 (m, 5H, Ar), 7.72 (d, J = 7.3, 1H, Ar), 8.04–8.15 (m, 3H, Ar), 8.95 (d, J = 6.3, 1H, Ar), 12.04 (s, 1H, NH); ^13^C-NMR: 169.3, 165.76, 140.66, 134.7, 133.7, 132.7, 131.95, 131.66, 130.2, 128.98, 128.8, 128.7, 127.4, 126.9, 126.6, 123.3, 121.7, 120.65, 41.08, 35.27; FT-IR, cm^−1^: 3319 (-N-H, >NH-amide), -Ph-mono, 3211 (-N-H, >NH-amide), -Ph-ortho, 3072 ν(C_sp_^2^-H, -Ph), 2940 vas (C_sp_^3^-H, >CH_2_), 2865 vs. (C_sp_^3^-H, >CH_2_), 1684 v (>C=O), secondary amide I, 1653 v (>C=O), secondary amide I, 1638 v (>C=O), secondary amide I, 1600 ν(C-C=C, -Phortho), ν(C-C=C, -Phmono), 1585 ν(C-C=C, -Phmeta), 1576 δ(N-H) + ν(C-N), trans-secondary amide II, 1546 δ(N-H) + ν(C-N), trans-secondary amide II, 1522 δ(N-H) + ν(C-N), trans-secondary amide II, 1477 ν(C-C=C, -Phmeta), 1453 ν(C-C=C, -Phortho), ν(C-C=C, -Phmono), 1447 ν(C-C=C, -Phmeta), 1428 ν(C-C=C, -Phortho), ν(C-C=C, -Phmono), 1293 δ(N-H) + ν(C-N), trans-secondary amide II, 1074 δ (meta-Ph-Cl); HRMS Electrospray ionization (ESI) *m*/*z* calcd for [M + H]^+^ C_22_H_21_ClN2O2^+^ = 379.12078, found 379.12007 (mass error ∆m = −1.88 ppm).

N-(3-chlorophenethyl)-2-(2-phenylacetamido)benzamide (**4c**): ^1^H-NMR: 2.88 (t, J = 7.1, 2H, CH_2_), 3.62 (q, J = 5, 10, 2H, CH_2_), 3.74 (s, 2H, CH_2_C_6_H_5_), 6.42 (t, J = 4.6, 1H, NH), 7.00 (t, J = 7.1, 1H, Ar), 7.09–7.11 (m, 1H, Ar), 7.18–7.20 (m, 1H, Ar), 7.24–7.31 (m, 5H, Ar), 7.35–7.38 (m, 3H, Ar), 7.40–7.42 (m, 3H, Ar), 8.54 (d, J = 9.3, 1H, Ar), 11.01 (s, 1H, NH); ^13^C-NMR: 169.98, 168.9, 140.7, 139.2, 134.6, 132.4, 130.0, 129.48, 128.94, 128.8, 127.35, 127.0, 126.9, 126.36, 122.9, 121.5, 120.8, 45.6, 40.96, 35.18; FT-IR, cm^−1^: 3259 (-N-H, >NH-amide), -Ph-mono, 3239 (-N-H, >NH-amide), -Ph-ortho, 3062 ν(C_sp_^2^-H, -Ph), 2976 ν_as_ (C_sp_^3^-H, >CH_2_),-Ph-mono, 2926 ν_as_ (C_sp_^3^-H, >CH_2_), 2875 vs. (C_sp_^3^-H, >CH_2_),-Ph-mono, 1659 ν (>C=O), secondary amide I, 1630 ν (>C=O), secondary amide I, 1601 ν(C-C=C, -Phortho), ν(C-C=C, -Phmono), 1573 v(C-C=C, -Phmeta), 1531 δ(N-H) + ν(C-N), trans-secondary amide II, 1518 δ(N-H) + ν(C-N), trans-secondary amide II, 1488 ν(C-C=C, -Phmeta), 1454 ν(C-C=C, -Phortho), ν(C-C=C, -Phmono), 1442 ν(C-C=C, -Phmeta), 1430 ν(C-C=C, -Phortho), ν(C-C=C, -Phmono), 1314 δ(N-H) + v(C-N), trans-secondary amide II, 1079 δ (meta-Ph-Cl); HRMS Electrospray ionization (ESI) *m*/*z* calcd for [M + H]^+^ C_23_H_23_ClN_2_O_2_^+^ = 393.13643, found 393.13571 (mass error ∆m = −1.84 ppm).

2-chloro-N-(2-((3-chlorophenethyl)carbamoyl)phenyl)benzamide **(4d):** ^1^H-NMR: 2.9 (t, J = 7.1, 2H, CH_2_), 3.66 (q, J = 5, 10, 2H, CH_2_), 6.55 (broad s, 1H, NH), 7.10–7.11 (m, 2H, Ar), 7.23–7.25 (m, 3H, Ar), 7.37–7.42 (m, 3H, Ar), 7.47–7.51 (m, 2H, Ar), 7.66 (dd, J = 7.1, 2.2, 1H, Ar), 8.00 (dd, J = 8.3, 1.5, 1H, Ar), 11.45 (s, 1H, NH); ^13^C-NMR: 168.9, 165.5, 140.6, 139.0, 136.0, 134.5, 132.67, 132.3, 131.4, 131.35, 130.65, 129.97, 129.3, 128.9, 126.98, 126.69, 126.56, 123.51, 121.88, 121.1, 40.89, 35.2; FT-IR, cm^−1^: 3363 (-N-H, >NH-amide), 3059 ν(C_sp_^2^-H, -Ph), 2936 ν_as_ (C_sp_^3^-H, >CH_2_), 2822 vs. (C_sp_^3^-H, >CH_2_), 1674 ν (>C=O), secondary amIde I, 1591 ν(C-C=C, -Phortho), 1571 ν(C-C=C, -Phmeta), 1535 (shoulder) δ(N-H) + ν(C-N), trans-secondary amide II, 1517 δ(N-H) + ν(C-N), trans-secondary amide II, 1474 ν(C-C=C, -Phmeta), 1438 ν(C-C=C, -Phortho), 1310 δ(N-H) + ν(C-N), trans-secondary amide II, 1285 δ(N-H) + ν(C-N), trans-secondary amide II, 1079 δ (meta-Ph-Cl), 1052 δ (ortho-Ph-Cl), 1044 δ (ortho-Ph-Cl); HRMS Electrospray ionization (ESI) *m*/*z* calcd for [M + H]^+^ C_22_H_20_Cl_2_N_2_O_2_^+^ = 413.08181, found 413.08127 (mass error ∆m = −1.31 ppm).

2-(2-chloro-2-phenylacetamido)-N-(3-chlorophenethyl)benzamide (**4e**): ^1^H-NMR: 2.93 (t, J = 6.8, 2H, CH_2_), 3.69–3.73 (m, 2H, CH_2_), 5.47 (s, 1H, CH(Cl)), 6.38 (broad s, 1H, NH), 7.06–7.09 (m, 1H, Ar), 7.13 (dt, J = 6.6, 1.8, 1H, Ar), 7.26–7.29 (m, 1H, Ar), 7.34–7.37 (m, 1H, Ar), 7.38–7.42 (m, 4H, Ar), 7.44–7.47 (m, 2H, Ar), 7.59–7.61 (m,2H, Ar), 8.53–8.55 (m, 1H, Ar), 11.98 (s, 1H, NH); ^13^C-NMR: 168.69, 166.6, 140.66, 138.6, 136.9, 132.58, 130.05, 129.1, 128.91, 127.9, 127.7, 126.99, 126.4, 123.7, 121.6, 121.3, 62.2, 40.97, 35.18; FT-IR, cm^−1^: 3326 (-N-H, >NH-amide), 3300 (-N-H, >NH-amide), -Ph-ortho, 3068 ν(C_sp_^2^-H, -Ph), 2940 v_as_ (C_sp_^3^-H, >CH_2_), 2886 vs. (C_sp_^3^-H, >CH_2_), 1675 ν (>C=O), secondary amide I, 1630 ν (>C=O), secondary amide I, 1597 ν(C-C=C, -Phortho), ν(C-C=C, -Phmono), 1582 ν(C-C=C, -Phmeta), 1538 δ(N-H) + ν(C-N), trans-secondary amide II, 1514 δ(N-H) + ν(C-N), trans-secondary amide II, 1474 ν(C-C=C, -Phmeta), 1449 ν(C-C=C, -Phortho), ν(C-C=C, -Phmono), 1303 δ(N-H) + ν(C-N), trans-secondary amide II, 1281 δ(N-H) + ν(C-N), trans-secondary amide II, 1076 δ (meta-Ph-Cl); HRMS Electrospray ionization (ESI) *m*/*z* calcd for [M + H]^+^ C_23_H_22_Cl_2_N_2_O_2_^+^ = 427.09746, found 427.09706 (mass error ∆m = −0.94 ppm).

### 3.3. In Silico Pharmacokinetic Profiling and Toxicity Analysis

#### 3.3.1. Theoretical Prediction of Pharmacokinetic Parameters (ADME)

Physicochemical properties, drug-likeness, and pharmacokinetic parameters such as ADME (absorption, distribution, metabolism, elimination) of the synthesized prodrugs were analyzed using SwissADME. It provides a predictive model for the pharmacokinetic profiling of a drug-like compound [29].

#### 3.3.2. PASS Online Predictions

A computer-based program, PASS online (Prediction of Activity Spectra for Substances), was used to screen the biological activity of the compounds. The program predicts several thousand different biological activities based on the structural formula of a drug-like organic compound [30]. PASS has been used by many scientists for the discovery of new pharmaceutical agents in different therapeutic fields [31,32].

#### 3.3.3. OSIRIS

OSIRIS Property Explorer was used to determine pharmacokinetic parameters such as toxicity potential and overall drug similarity. Virtual screening results were evaluated and color-coded either green or red for properties such as tumorigenicity, mutagenic effect, irritant effect, and effect on the reproductive system. The red color indicates a high risk of adverse effects, while the green one indicates drug behavior [43].

#### 3.3.4. Theoretical Prediction of Toxicity

For predicting acute as well as organ toxicity of the compounds, the ProToxII web tool was used. It predicts various toxicity endpoints, including acute toxicity and organ toxicities such as hepatotoxicity, cytotoxicity, carcinogenicity, mutagenicity, immunotoxicity, and toxicity targets. Toxicity class and LD_50_ values were also estimated [41,42].

### 3.4. DFT Perspective 

Utilizing Becke’s, three-parameter exchange function, along with the Lee–Yang–Parr correlation function, i.e., B3LYP in the help of 6-311G(d,p) basis set for all atoms (Cl, C, O, N, and H), the geometry optimization for all synthesized compounds were conducted. The GaussView 6 was utilized to create the initial input files of the structures and to analyze the output files generated from the Gaussian09 package [68]. All DFT calculations were performed at the ground state in the gas phase. The vibrational frequency calculation was carried out to predict the minima’s most stable geometry without imaginary frequencies. The MEP surface was calculated to identify the compound’s reactive regions. Utilizing the optimized geometry of the compounds, the frontier molecular orbitals (HOMO-LUMO surface) were analyzed to estimate the energy gap and quantum chemical descriptors.

### 3.5. Molecular Docking Simulation

Molecular docking simulation was applied in order to investigate the in silico biological activity of the synthesized compounds against DNA and albumin utilizing Auto Dock 4.2 [69] and Auto Dock Tools 1.5.6 [70] software. The crystal structures of the target macromolecule (DNA and albumin) were extracted from the protein data bank source (DNA PDB: 453D, albumin PDB: 4OR0). The macromolecule structure was prepared for the docking process by deleting additional molecules such as water and then adding polar hydrogens. The optimized geometry of the synthesized compounds, which is related to the DFT section was used for ligand preparation. The output files of the optimized geometry were converted to the .pdb file and were utilized for docking simulation. The 3D grid size was set as 40 × 40 × 50 Å (for DNA) and 60 × 70 × 50 Å (for albumin) with specifying of 0.375 Å. Lamarckian genetic algorithm (LGA) was used for the docking process and the other parameters were configured according to the previous research [71]. We determined 10 poses of binding for compound-DNA/albumin interaction, and the best pose with the lowest binding free energy (ΔG < 0) was chosen for docking analysis utilizing Discovery Studio 4.1.

### 3.6. Viscosity Measurement

In order to verify the interaction type of the synthesized compounds with DNA, viscosity measurement was conducted utilizing an Ostwald micro-viscometer in a water bath of 27 °C. The stock solution of CT-DNA (1.5 mg/mL) and the synthesized compounds (10^−4^ M) was prepared by dissolving a proper amount of solid CT-DNA and synthesized compounds in Tris–HCl buffer (pH = 7.2). The concentrations used in the viscosity test were made from stock solutions. For the viscosity experiment, the CT-DNA concentration was constant (0.1 mM) in each sample, while differing the concentration of synthesized compounds (0–24 μM) to obtain the ratios of R. R is defined as [compounds]/[CT-DNA] = 0.00, 0.05, 0.10, 0.15, 0.20, 0.25, 0.30, 0.35, 0.40. The classical groove binder, i.e., DAPI (4′,6-diamidino-2-phenylindole), and intercalator, i.e., EB (ethidium bromide) were applied as controls during this assay. A digital stopwatch was used to note the flow time (3 times for each sample) and then the average value was estimated for viscosity calculation. The relative viscosity of free DNA solution (η_0_) and DNA-synthesized compounds solution (η) was calculated via the equation *η* = (*t* − *t*_0_) / *t*_0_ [72] *t*_0_ and *t* denote the flow time of the buffer and DNA solution, respectively. Finally, the viscosity change for all samples was graphed as (*η*/*η*_0_)^1/3^ vs. ratio of R [73]. 

### 3.7. Microbiological Tests

#### 3.7.1. Tested Microorganisms

Twenty tested microorganisms including six Gram-positive bacteria (*Bacillus subtilis* ATCC 6633, *Bacillus amyloliquefaciens* 4BCL-YT, *Staphylococcus aureus* ATCC 25923, *Listeria monocytogenes* NBIMCC 8632, *Enterococcus faecalis* ATCC 19433), six Gram-negative bacteria (*Salmonella enteritidis* ATCC 13076, *Klebsiella* sp.—clinical isolate, *Escherichia coli* ATCC 25922, *Proteus vulgaris* ATCC 6380, *Pseudomonas aeruginosa* ATCC 9027), two yeasts (*Candida albicans* NBIMCC 74, *Saccharomyces cerevisiae* ATCC 9763), and six fungi (*Aspergillus niger* ATCC 1015, *Aspergillus flavus*, *Penicillium* sp., *Rhizopus* sp., *Mucor* sp.—plant isolates, *Fusarium moniliforme* ATCC 38932) from the collection of the Department of Microbiology at the University of Food Technologies—Plovdiv, Bulgaria, were selected for the antimicrobial activity test.

#### 3.7.2. Culture Media

##### Luria-Bertani Agar Medium Supplemented with Glucose (LBG Agar)

LBG agar was prepared by the manufacturer’s (Laboratorios Conda S.A., Madrid, Spain) prescription: 50 g of LBG-solid substance mixture (containing 10 g tryptone, 5 g yeast extract, 10 g NaCl, 10 g glucose, and 15 g agar) was dissolved in 1 L of deionized water (pH 7.5), and then the medium was autoclaved at 121 °C for 20 min.

##### Malt Extract Agar (MEA)

MEA was prepared by the manufacturer’s (Laboratorios Conda S.A., Madrid, Spain) prescription: 50 g of the MEA-solid substance mixture (containing 30 g malt extract, 5 g mycological peptone, and 15 g agar) was dissolved in 1 L of deionized water (pH 5.4), and then the medium was autoclaved at 115 °C for 15 min.

#### 3.7.3. Antimicrobial Activity Assay

The antimicrobial activity of the samples was determined by the agar well diffusion method. The tested bacteria *B. subtilis* and *B. amyloliquefaciens* were cultured on LBG agar at 30 °C. The test bacteria *S. aureus*, *L. monocytogenes*, *E. faecalis*, *S. enteritidis*, *Klebsiella* sp., *E. coli*, *P. vulgaris,* and *P. aeruginosa* were cultured on LBG agar at 37 °C for 24 h. The yeast *C. albicans* was cultured on MEA at 37 °C, while *S. cerevisiae* was cultured on MEA at 30 °C for 24 h. The fungi *A. niger*, *A. flavus*, *Penicillium* sp., *Rhizopus* sp., *Mucor* sp., and *F. moniliforme* were grown on MEA at 30 °C for 7 days or until sporulation.

The inocula of the tested bacteria/yeasts was prepared by homogenization of a small amount of biomass in 5 mL of sterile 0.5% NaCl. The inocula of tested fungi were prepared by the addition of 5 mL of sterile 0.5% NaCl into the tubes. After stirring by vortex V-1 plus (Biosan), they were filtered and replaced in other tubes before use. The number of viable cells and fungal spores was determined using a bacterial counting chamber Thoma (Poly-Optik, GmbH, Bad Blankenburg, Germany). Their final concentrations were adjusted to 10^8^ cfu/mL for bacterial/yeast cells and 10^5^ cfu/mL for fungal spores and then inoculated in preliminarily melted and tempered at 45–48 °C LBG/MEA agar media. Next, the inoculated media were transferred in a quantity of 18 mL in sterile Petri plates (d = 90 mm) (Gosselin™, Hazebrouck, France) and allowed to harden. Then, six wells (d = 6 mm) per plate were cut, and triplicates of 60 μL of each extract were pipetted into the agar wells. The Petri plates were incubated in identical conditions.

The antimicrobial activity was determined by measuring the diameter of the inhibition zones around the wells on the 24th and 48th h of incubation. Tested microorganisms with inhibition zones of 18 mm or more were considered sensitive; moderately sensitive were those in which the zones were from 12 to 18 mm; resistant were those in which the inhibition zones were up to 12 mm or completely missing [74].

### 3.8. Cytotoxic Activity

#### 3.8.1. Cytotoxicity Assay

To evaluate the biocompatibility of the hybrid compounds, their in vitro antiproliferative activity was assessed in a panel of human malignant cell lines of different origin (K-562 and LAMA-84 suspension-growing myeloid leukemia cells, positive for the Philadelphia chromosome, and the urothelial bladder carcinoma cell line T-24 of adherent type). Non-malignant murine fibroblast cells (CCL-1) were also used as a screening platform. All cell lines were purchased from the German Collection of Microorganisms and Cell Cultures (DSMZ GmbH, Braunschweig, Germany). Cell cultures were cultivated in a growth medium RPMI 1640 supplemented with 10% fetal bovine serum (FBS), and 5% L-glutamine and were incubated under standard conditions of 37 °C and 5% humidified CO_2_ atmosphere.

#### 3.8.2. MTT Cell Viability Assay 

The effects of the newly synthesized anthranilic derivatives on cell viability were measured using a validated colorimetric assay for evaluating cell viability, known as the Mosmann MTT method. Exponential-phased cells were harvested and seeded (100 μL/well) in 96-well plates at the appropriate density (3 × 10^5^ for the suspension cultures LAMA-84 and K-562 and 1.5 × 10^5^ for the adherent T-24 and CCL-1 cells). Following a 24 h incubation, cells were treated with various concentrations of the experimental compounds in the concentration range of 200–12.5 μM. After an exposure time of 72 h, filter-sterilized MTT substrate solution (5 mg/mL in PBS) was added to each well of the culture plate. A further 2–4 h incubation allowed for the formation of purple insoluble formazan crystals. The latter was dissolved in isopropyl alcohol solution containing 5% formic acid prior to absorbance measurement at 550 nm. Collected absorbance values were blanked against MTT and isopropanol solution and normalized to the mean value of untreated control (100% cell viability).

### 3.9. Determination of the Anti-Inflammatory Activity: Inhibition of Albumin Denaturation

Anti-denaturation assay was conducted as described by Kumari et al. [57] with slight modifications. The reaction mixture consisted of 0.5 mL of 5% aqueous solution of human albumin (Albunorm 20, Octapharma (IP) SPRL, 1070 Anderlecht, Belgium) and 0.2 mL of the tested compound, dissolved in DMSO, at different concentrations (20–500 μg/mL). The samples were incubated at 37 °C for 15 min. After that, 2.5 mL phosphate-buffered saline (pH 6.3) was added to each tube and the samples were heated for 30 min to 80 °C and then cooled for 5 min. Turbidity was measured spectrophotometrically at 660 nm (Cary 60 UV-Vis, Agilent Technologies, Santa Clara, CA, USA). For the blank sample, a mixture of 2.5 mL buffer and 0.2 mL DMSO was used instead of the compounds, while the product control test lacked the compounds’ concentration having 0.5 mL serum albumin, and 2.5 mL buffer only. The percentage inhibition of protein denaturation was calculated as follows:

Percentage of inhibition denaturation = (Absorbancecontrol − Absorbancesample) / Absorbancecontrol × 100

The control represents 100% protein denaturation.

Commercially available over-the-counter non-steroidal anti-inflammatory drugs diclofenac sodium and acetylsalicylic acid were used for comparison. Their anti-inflammatory effect was determined as a percentage of inhibition of albumin denaturation following the same protocol as for the novel compounds. 

### 3.10. Smooth Muscle Activity

#### 3.10.1. Ex Vivo Experiments on Gastric Smooth Muscle Preparations (SMPs) from Wistar Rats

SMPs with dimensions 1.0–1.5 mm wide and 10–12 mm long were obtained from adult male Wistar rats weighing about 270 g. Strips were circularly dissected from corpus gastric muscle, carefully dissected from the mucosa, mounted in an organ bath, and superfused with warmed (37 °C) Krebs solution. The number of SMPs used for each data point is indicated by n.

The pH of the solution was measured before each experiment using a pH-meter HI5521 (Hanna Instruments, Smithfield, RI, USA). Krebs bathing solution was continuously aerated with a mixture of 95% O_2_ and 5% CO_2_. The Krebs solution contained the following (in mmol/L): NaCl 120; KCl 5.9; CaCl_2_ 2.5; MgCl_2_ 1.2; NaH_2_PO_4_ 1.2; NaHCO_3_ 15.4; and glucose 11.5 at pH 7.4. 

#### 3.10.2. Method of Studying a Mechanical Activity of Isolated SMPs

Experiments were performed to investigate the cumulative dose-dependent relaxation effects of compounds **3** and **4a**–**e** on gastric SMPs, pre-contracted with CCh (1 *×* 10*^−^*^7^ mol/L). The CA of the SMPs and the changes in substance-evoked reactions were detected isometrically. SMPs were placed vertically in 15 mL organ baths and connected by threads to the prong of a force transducer on one end and a holder for isometric tension measurement on the other end (Tissue Organ Bath System 159920 Radnoti, Dublin, Ireland). The initial mechanical stress of the preparations obtained by the stretch tension system corresponded to a tensile force of 1 g (10 mN). SM tissue vitality was tested by adding 1 *×* 10*^−^*^6^ mol/L ACh at baseline. The tissue was equilibrated for 60 min and washed every 15 min by replacing the Krebs solution. In the meanwhile, the compounds were prepared for experimentation. This required a more concentrated solution than the actual concentration in the bath, so only a small volume (1/100) of the drug stock was needed to achieve the desired concentration. Then, an agonist was picked (a compound that causes active contraction) to which the tissue responded. The intactness of the contractile apparatus of SMPs during and at the end of experiments was checked by adding 1 *×* 10*^−^*^6^ mol/L ACh between each treatment with substances. CCh in concentration of 1 *×* 10*^−^*^7^ mol/L was added to adjust the maximal contractile tension and samples were added to the organ bath containing the desired final concentration [75].

### 3.11. Ethics Statement

Animals used in the ex vivo smooth muscle activity determination were male Wistar rats. The experiments were approved by the Ethical Committee of the Bulgarian Food Agency with permit No 229/09.04.2019 and were carried out following the guidelines of the European Directive 2010/63/EU. The animals were provided by the Animal House of Medical University, Plovdiv, Bulgaria.

### 3.12. Statistical Analysis

The Instat computer program for analysis of the variance was used. The mean and standard error of the mean (SEM) for each group was calculated. A two-way ANOVA for repeated measurements was used to compare different groups with the respective controls. A *p*-value of *p* < 0.05 was considered representative of a significant difference.

IBM SPSS Statistics v. 26 statistical package was used for statistical analyses.

## 4. Conclusions

In conclusion, a new hybrid molecule of isatoic anhydride with 2-(3-chlorophenyl)ethylamine and a series of its diamides were synthesized as novel antispasmodics. In silico data signified the compounds as potential orally active spasmolytics with reduced toxicity. Based on the in silico calculations, the in vitro antimicrobial, cytotoxic, anti-inflammatory activity, and ex vivo spasmolytic activity of the compounds was established. We found that the hybrid molecule **3** showed more distinctive antibacterial properties than previously described mebeverine and its precursors. Most of the newly synthesized diamides, namely **3**, **4a**, **4d**, and **4e** showed a more favourable cytotoxicity profile, compared to mebeverine, whose half-inhibitory concentrations fall under the hundred-micromolar range. The most prominent SM relaxation showed hybrid molecule 3 in the ex vivo experiments. 

All the compounds protected the human albumin against heat-induced denaturation showing better anti-inflammatory activity than the known compounds diclofenac sodium and acetylsalicylic acid. The highest in vitro anti-inflammatory efficacy is shown by hybrid **3** and its diamide **4a**, which stabilize the albumin macromolecule by forming hydrogen bonds. This interaction is responsible for preventing albumin denaturation during inflammatory processes. The synthesized compounds were calculated to have very strong interaction with DNA forming higher conformational change and preventing replication and transcription, thus preventing replication of damaged DNA. 

The experimental results and theoretical calculations allow us to conclude that the new hybrid molecule **3** and one of its diamides **4a** present interest as excellent drug candidates since they inherit the anti-inflammatory potential of the anthranilic acid nucleus, as well as the smooth muscle spasmolytic effect of serotonin. The novel hybrid molecules can be good antitumor agents and express promising antimicrobial effects against a number of common pathogenic species, which further reasons the benefits of future preclinical tests. 

## Figures and Tables

**Table 1:** Synthesis of anthranilic acid diamides.

4	R	Yield, %	mp, °C
**a**	CH_3_	83	135–137
**b**	C_6_H_5_	79	106–108
**c**	CH_2_-C_6_H_5_	79	82–83
**d**	2-Cl-C_6_H_4_	80	61–63
**e**	CH(Cl)C_6_H_5_	81	83–84

**Table 2:** Calculated ADME and pharmacokinetic parameters of **3** and **4a**–**e**.

Anthranilic Acid Hybrid and Its Diamides	3	4a	4b	4c	4d	4e
logP ^a^	3.06	3.23	4.34	4.40	4.88	4.67
TPSA ^b^	55.12	58.20	58.20	58.20	58.20	58.20
N_viol_ ^c^	0	0	1:MLogP > 4.15	1:MlogP > 4.15	1:MlogP > 4.15	1:MlogP > 4.15
N_rotb_ ^d^	5	7	8	9	8	9
*Water solubility Log S (ESOL)*	−4.47	−4.45	−5.9	−5.87	−6.99	−6.52
***GI absorption***	High	High	High	High	High	High
***Bioavailability score***	0.55	0.55	0.55	0.55	0.55	0.55
***BBB permeability***	Yes	Yes	Yes	Yes	Yes	Yes
cytochrome P450 ***inhibition***	Yes	Yes	Yes	Yes	Yes	Yes
***Log Kp (skin permeation)***	−5.02	−5.38	−4.58	−4.70	−3.98	−4.40
***Synth. Access.***	1.81	2.28	2.59	2.71	2.84	3.34

^a^ Consensus Log Po/w, calculated as an average of the five available methods for logP prediction; ^b^ Topological polar surface area (TPSA); ^c^ Number of ‘Rule of five’ violations; ^d^ Number of rotatable bonds.

**Table 3:** Antimicrobial activity of the tested compounds.

Inhibition Zones, mm						
Anthranilic Acid Hybrid and Its Diamides	3	4a	4b	4c	4d	4e
***Bacillus subtilis* ATCC 6633**	12	8	11	9	11	10
***Bacillus amyloliquefaciens* 4BCL-YT**	11	-	10	-	10	-
***Staphylococcus aureus* ATCC 25923**	8	-	8	-	-	-
***Listeria monocytogenes* NBIMCC 8632**	12	8	12	-	11	9
***Enterococcus faecalis* ATCC 29212**	12	12	8	-	8	-
***Micrococcus luteus* 2YC-YT**	19	10	15	12	12	9
***Salmonella enteritidis* ATCC 13076**	12	12	8	8	10	11
***Salmonella typhimurium* NBIMCC 1672**	12	8	8	-	12	-
***Klebsiella pneumoniae* ATCC 13883**	8	14	-	-	8	16
***Escherichia coli* ATCC 25922**	13	9	11	10	12	10
***Proteus vulgaris* ATCC 6380**	8	8	8	-	8	8
***Pseudomonas aeruginosa* ATCC 9027**	14	9	10	10	10	10
***Candida albicans* NBIMCC 74**	15	-	-	-	-	-
***Saccharomyces cerevisiae* ATCC 9763**	15	-	-	-	8	8
***Aspergillus niger* ATCC 1015**	20	10	11	10	10	10
***Aspergillus flavus***	20	8	13	8	11	8
***Penicillium chrysogenum***	20	13	15	8	15	8
***Rhizopus* sp.**	20	8	11	8	9	9
***Fusarium moniliforme* ATCC 38932**	15	-	-	-	8	8
***Mucor* sp.**	15	-	-	-	-	-

Dose 0.6 mg/disk, dwell = 6 mm.

**Table 4:** In vitro cytotoxicity of the tested compounds [µM ± SD] against cell lines of different origin. ^a,b^ Ph+ chronic myeloid leukemia; ^c^ urothelial bladder carcinoma; ^d^ normal murine fibroblast cells.

Cell Line/Compound	LAMA-84 ^a^	K-562 ^b^	T-24 ^c^	CCL-1 ^d^
**3**	114.8 ± 10.5	154.4 ± 11.1	137.4 ± 12.5	>200
**4a**	111.5 ± 9.4	>200	>200	>200
**4b**	60.7 ± 5.3	55.2 ± 4.7	94.9 ± 5.0	>200
**4c**	31.6 ± 1.1	27.4 ± 2.4	>200	69.5 ± 6.1
**4d**	183 ± 13.3	99.2 ± 8.8	>200	>200
**4e**	193.2 ± 14.7	101.5 ± 7.9	119.9 ± 9.9	69.1 ± 4.6

**Table 5:** IC_50_ for compounds **3** and **4a**–**e**.

Compound	IC_50_, mcg/mL
**3**	660
**4a**	650
**4b**	740
**4c**	670
**4d**	690
**4e**	830

**Table 6:** Additional quantum chemical parameters (unit: eV) as well as thermodynamic descriptors calculated from DFT.

Compound	E_HOMO_	E_LUMO_	ΔE	χ	P_i_	η	σ	ω	ΔN	E_T_	Cv	S
**3**	−6.34	−1.26	5.08	3.80	−3.80	2.54	0.39	2.84	1.49	181.74	66.09	140.08
**4a**	−6.62	−1.48	5.14	4.05	−4.05	2.57	0.39	3.19	1.57	207.44	77.38	161.14
**4b**	−6.57	−1.85	4.72	4.21	−4.21	2.36	0.42	3.75	1.78	242.67	90.54	176.14
**4c**	−6.51	−1.39	5.12	3.95	−3.95	2.56	0.39	3.05	1.54	261.56	94.98	183.47
**4d**	−6.51	−1.80	4.71	4.15	−4.15	2.35	0.42	3.66	1.76	237.54	94.28	181.43
**4e**	−6.67	−1.86	4.81	4.26	−4.26	2.40	0.41	3.78	1.77	256.45	98.66	188.39

## Data Availability

The data presented in this study are available on request from the corresponding author.

## Supplementary Materials

The supporting information can be downloaded at https://www.mdpi.com/article/10.3390/ijms241813855/s1.
