# Supplementary material for: Synthesis, Molecular Docking, and Biological Evaluation of Novel Anthranilic Acid Hybrid and Its Diamides as Antispasmodics
Source: Int J Mol Sci. 2023 Sep 8;24(18):13855. doi: 10.3390/ijms241813855 (PMC10530836; doi:10.3390/ijms241813855)

**Synthesis, Molecular Docking, And Biological Evaluation Of Novel Anthranilic Acid Hybrid And Its Diamides**

Miglena Milusheva, Vera Gledacheva, Iliyana Stefanova, Mehran Feizi-Dehnayebi, Rositsa Mihaylova, Paraskev Nedialkov, Emiliya Cherneva, Yulian Tumbarski, Slava Tconeva, Mina Todorova, and Stoyanka Nikolova

**Table of Contents:**

Figure S1: $^{1}$H-NMR spectrum of compound **3**, page 2

Figure S2: $^{13}$C-NMR spectrum of compound **3**, page 3

Figure S3: DEPT spectrum of compound **3**, page 4

Figure S4: FT-IR spectrum of compound **3**, page 5

Figure S5: $^{1}$H-NMR spectrum of compound **4a**, page 6

Figure S6: $^{13}$C-NMR spectrum of compound **4a**, page 7

Figure S7: DEPT spectrum of compound **4a**, page 8

Figure S8: FT-IR spectrum of compound **4a**, page 9

Figure S9: $^{1}$H-NMR spectrum of compound **4b**, page 10

Figure S10: $^{13}$C-NMR spectrum of compound **4b**, page 11

Figure S11: DEPT spectrum of compound **4b**, page 12

Figure S12: FT-IR spectrum of compound **4b**, page 13

Figure S13: $^{1}$H-NMR spectrum of compound **4c**, page 14

Figure S14: $^{13}$C-NMR spectrum of compound **4c**, page 15

Figure S15: DEPT spectrum of compound **4c**, page 16

Figure S16: FT-IR spectrum of compound **4c**, page 17

Figure S17: $^{1}$H-NMR spectrum of compound **4d**, page 18

Figure S18: $^{13}$C-NMR spectrum of compound **4d**, page 19

Figure S19: DEPT spectrum of compound **4d**, page 20

Figure S20: FT-IR spectrum of compound **4d**, page 21

Figure S21: $^{1}$H-NMR spectrum of compound **4e**, page 22

Figure S22: $^{13}$C-NMR spectrum of compound **4e**, page 23

Figure S23: DEPT spectrum of compound **4e**, page 24

Figure S24: FT-IR spectrum of compound **4e**, page 25

Figure S1: $^{1}$H-NMR spectrum of compound **3**

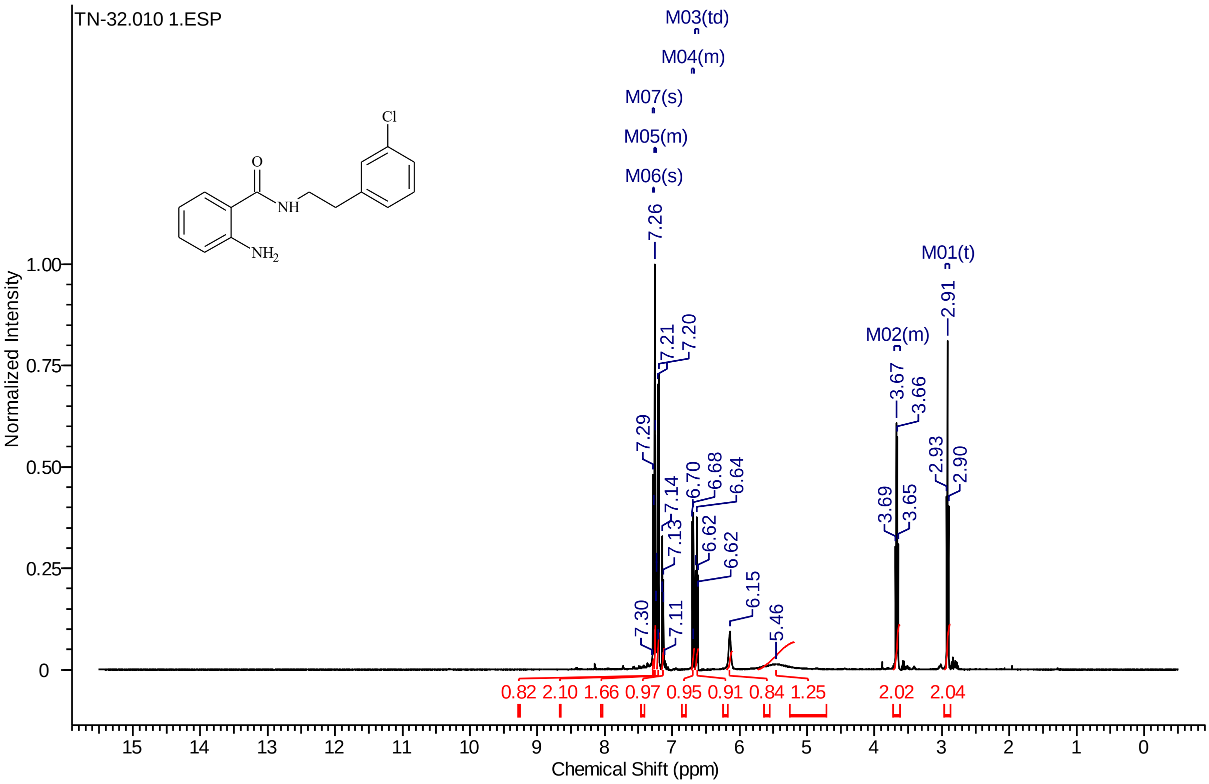

Figure S2: $^{13}$C-NMR spectrum of compound **3**

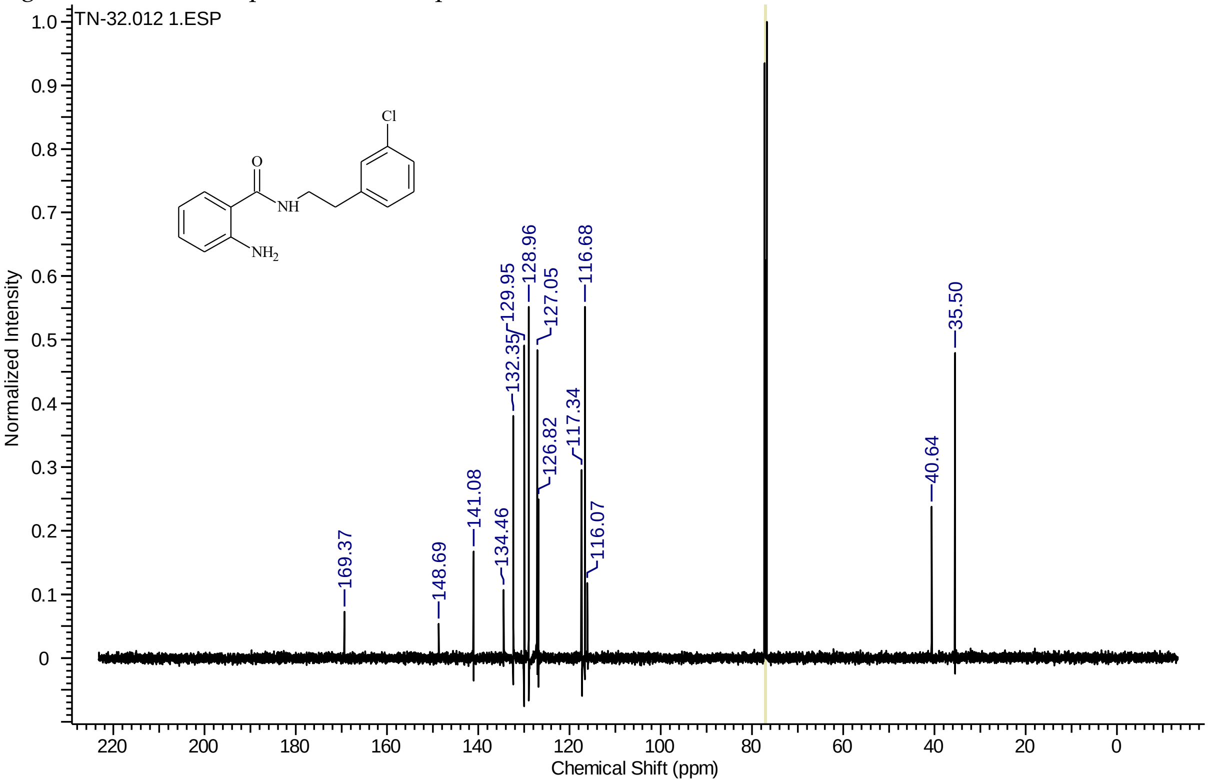

Figure S3: DEPT spectrum of compound **3**

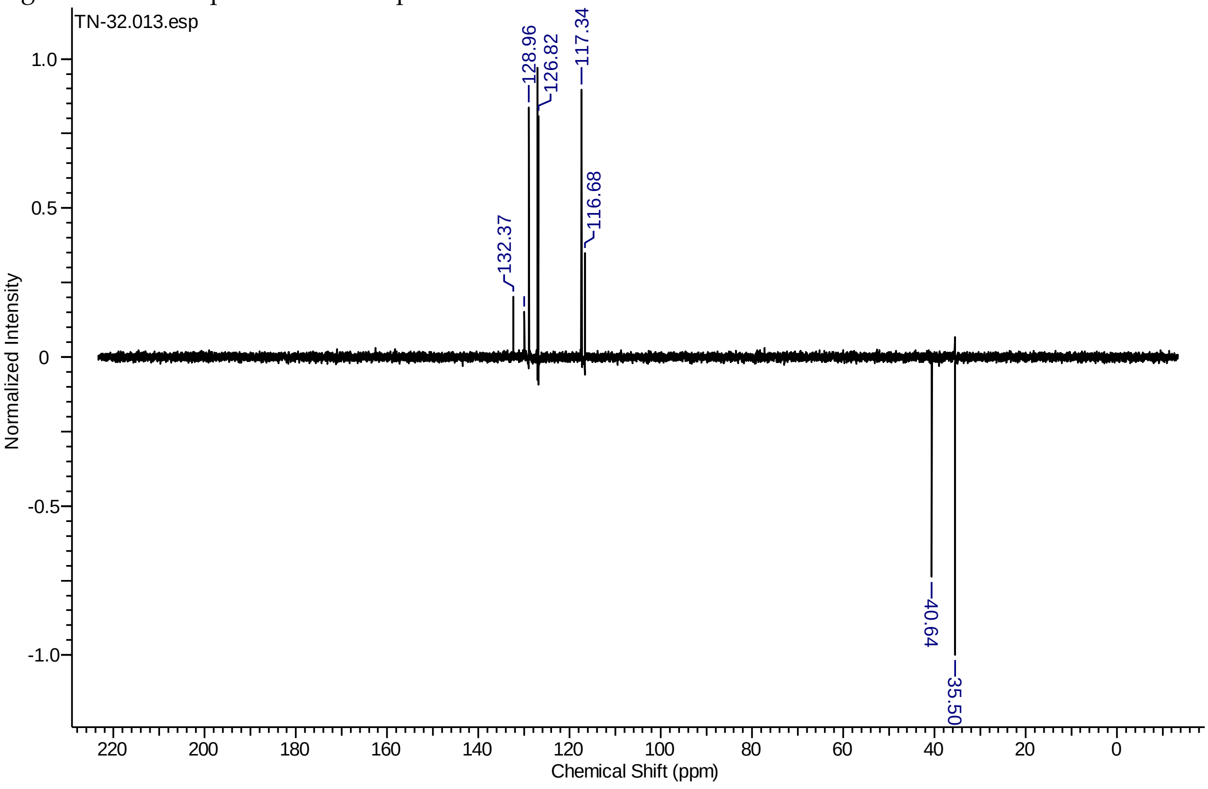

Figure S4: FT-IR spectrum of compound **3**

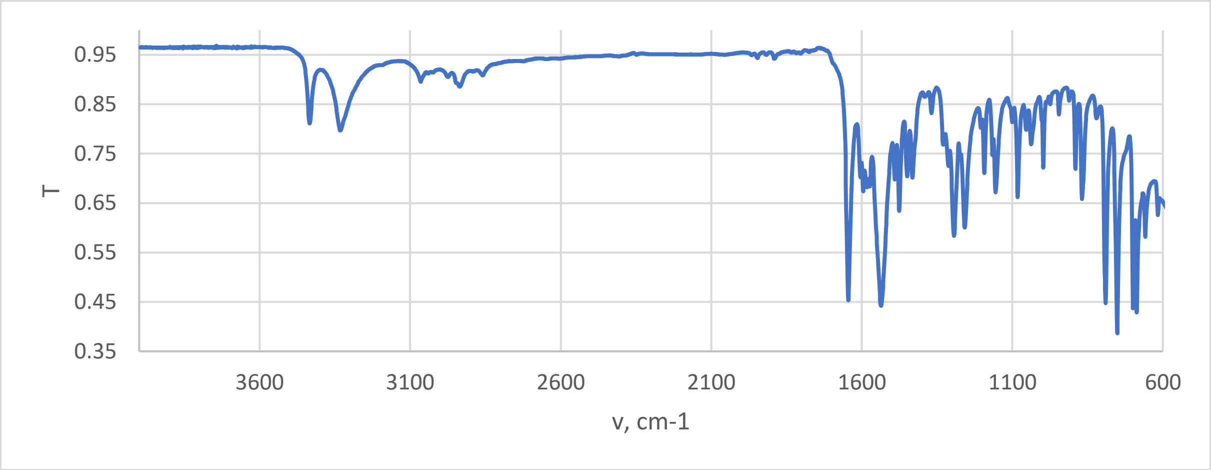

Figure S5: $^{1}$H-NMR spectrum of compound **4a**

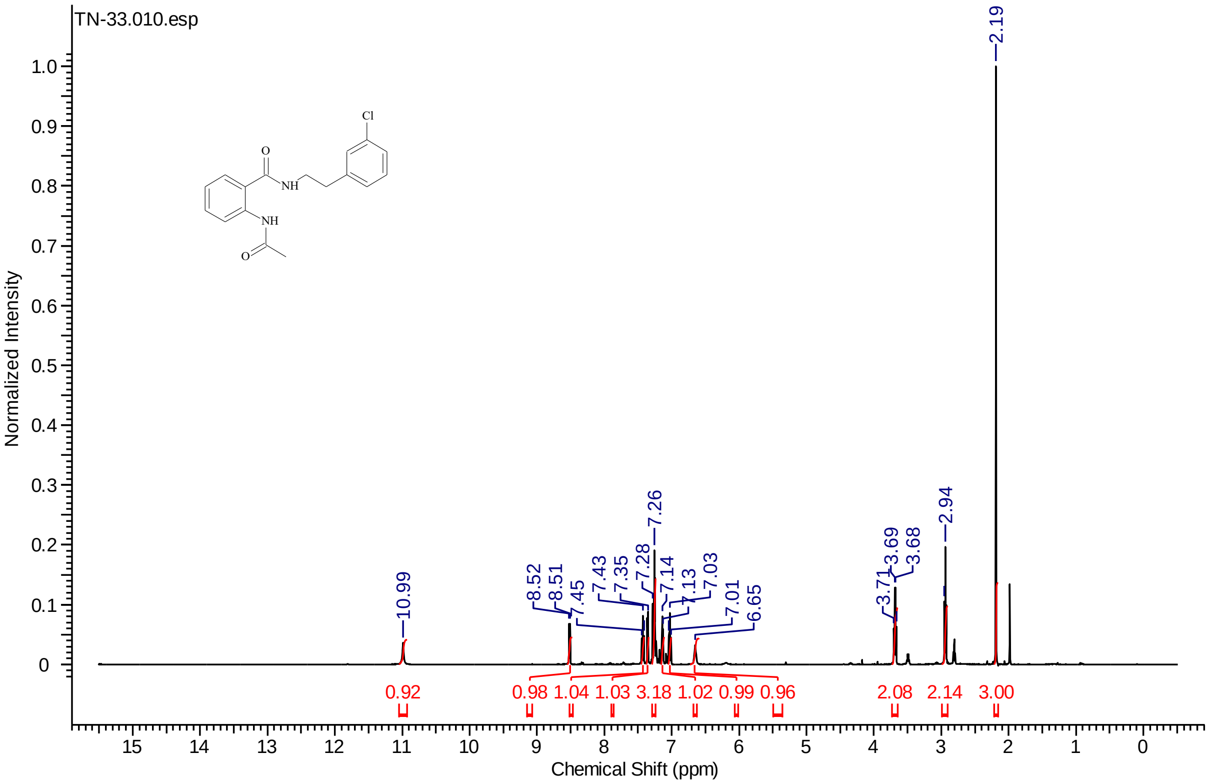

Figure S6: $^{13}$C-NMR spectrum of compound **4a**

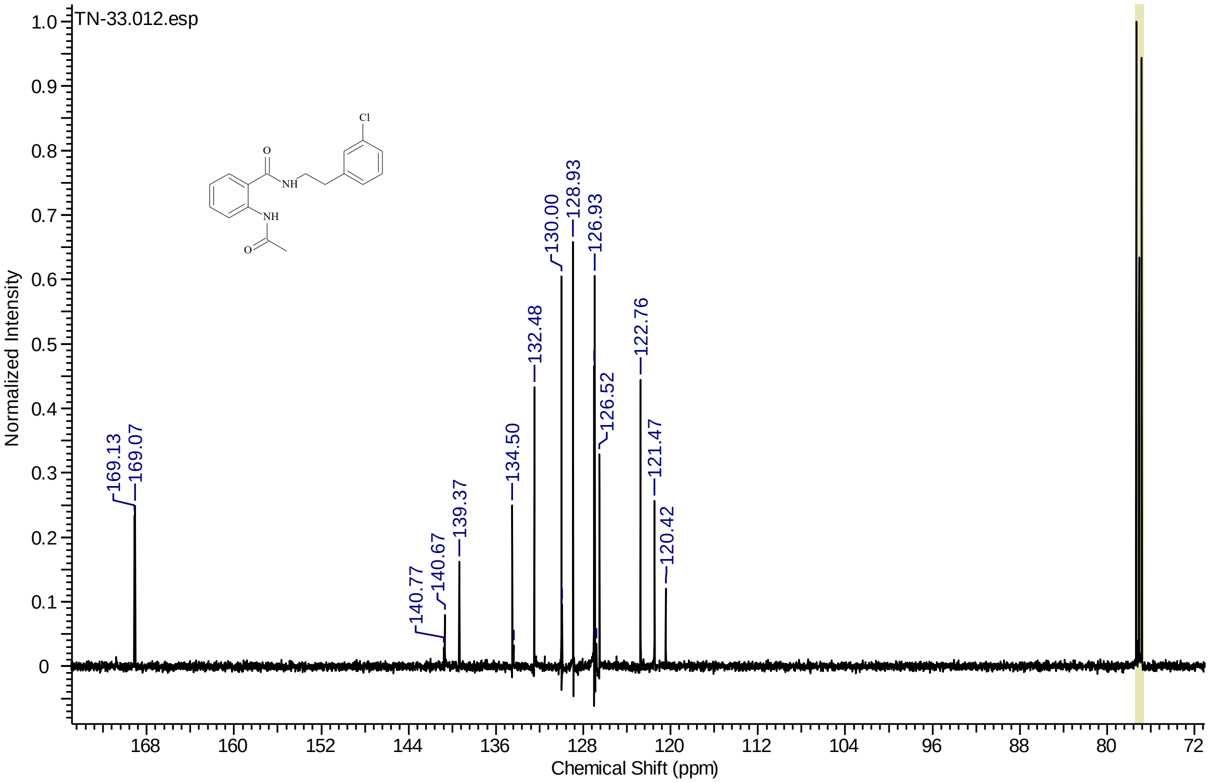

Figure S7: DEPT spectrum of compound **4a**

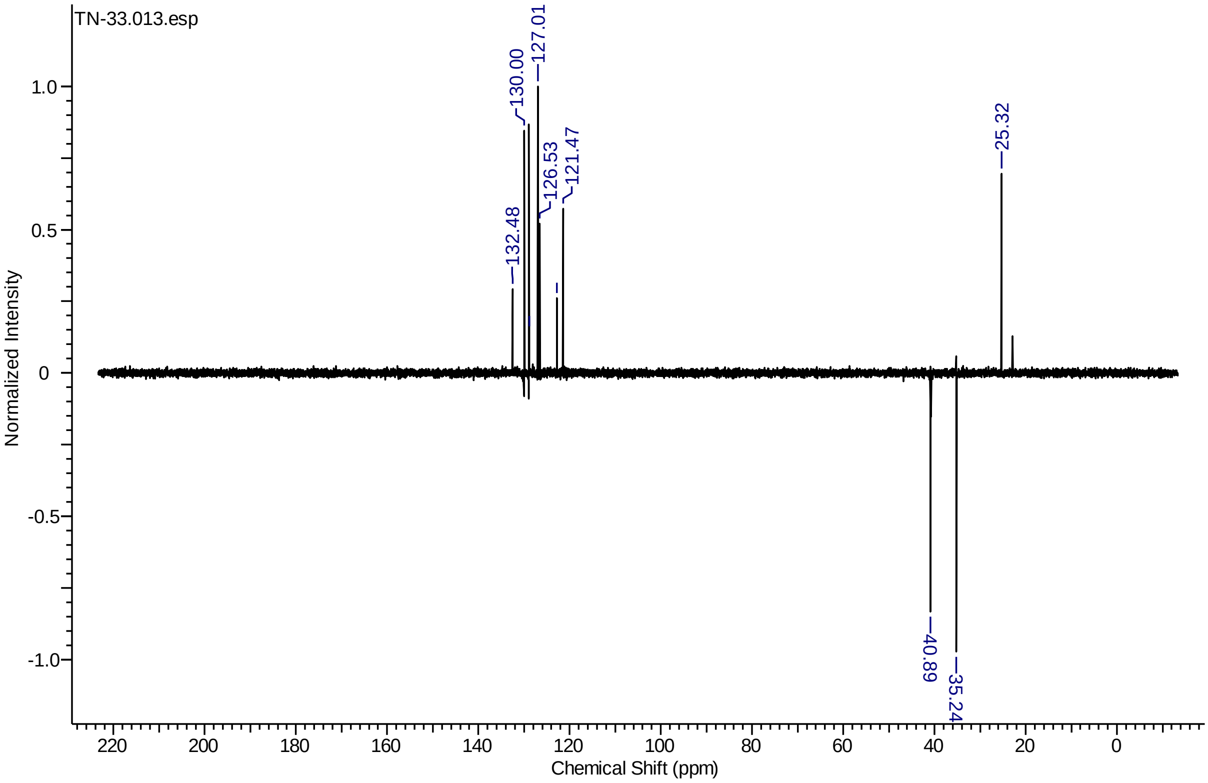

Figure S8: FT-IR spectrum of compound **4a**

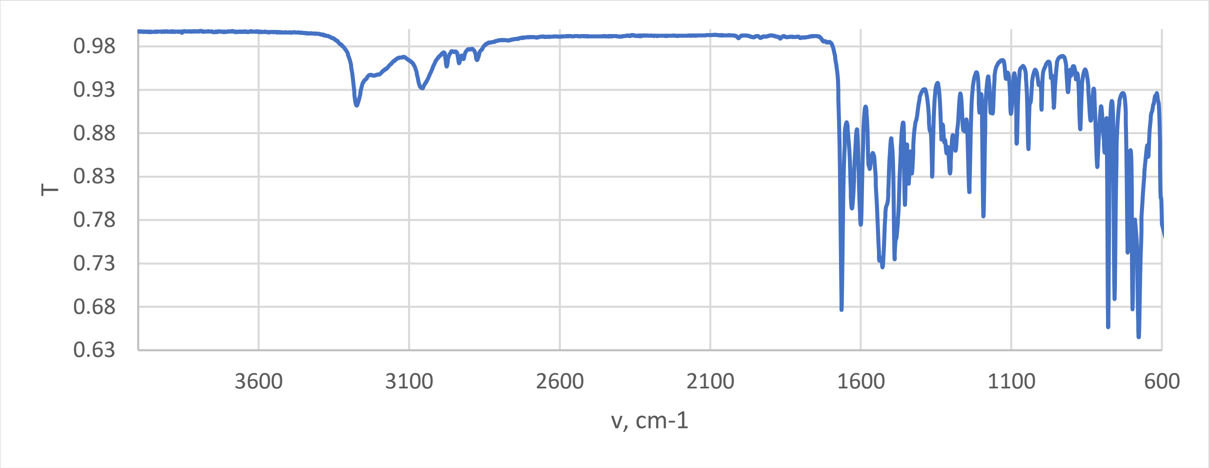

Figure S9: $^{1}$H-NMR spectrum of compound **4b**

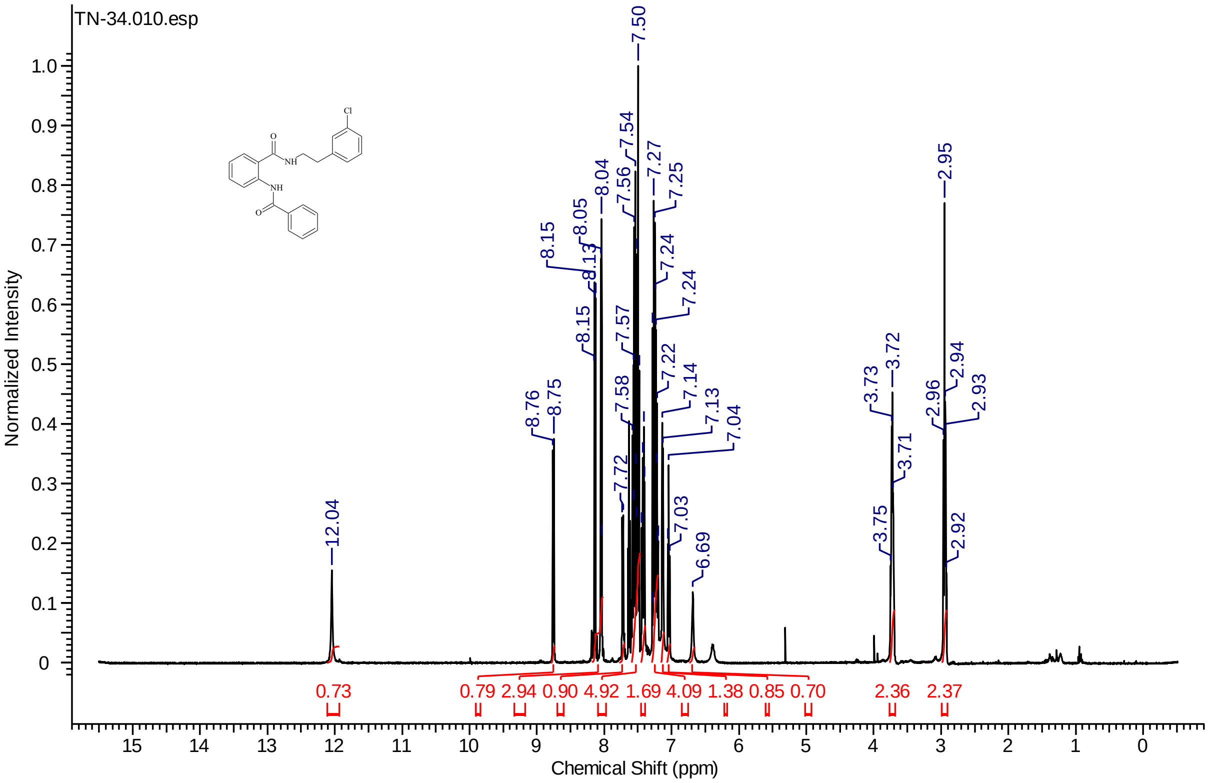

Figure S10: $^{13}$C-NMR spectrum of compound **4b**

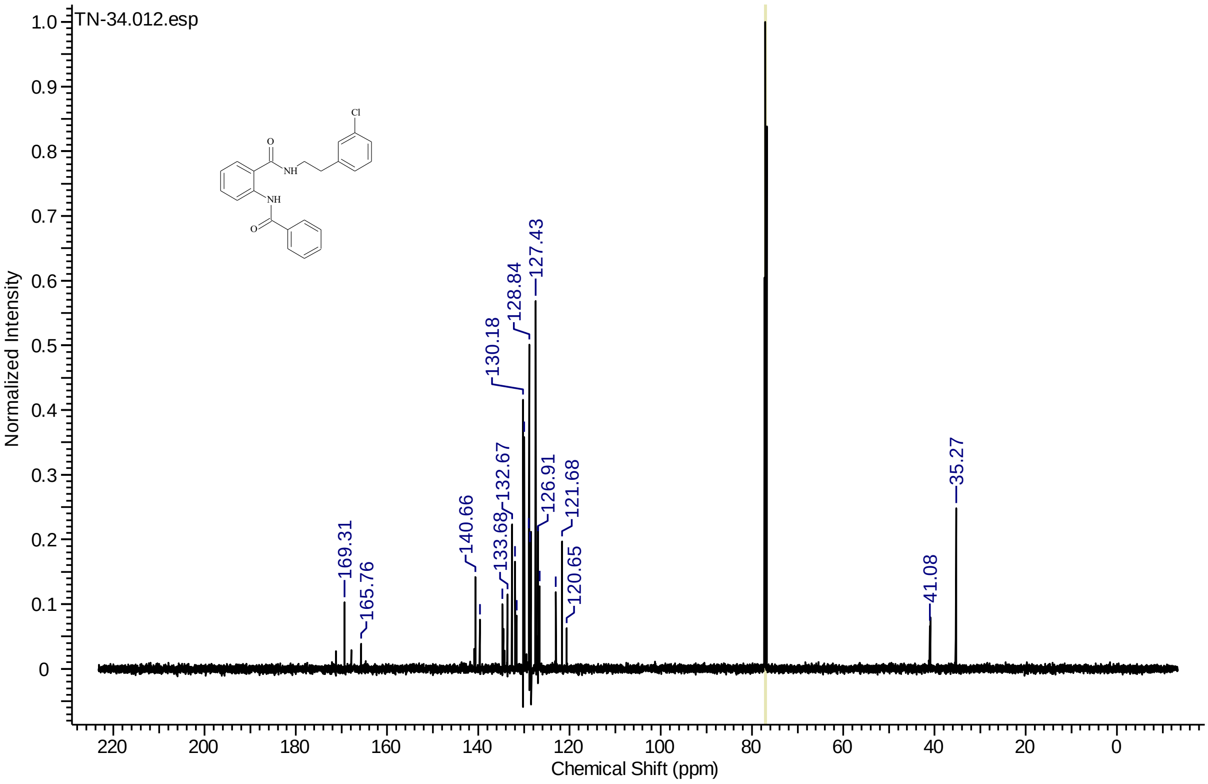

Figure S11: DEPT spectrum of compound **4b**

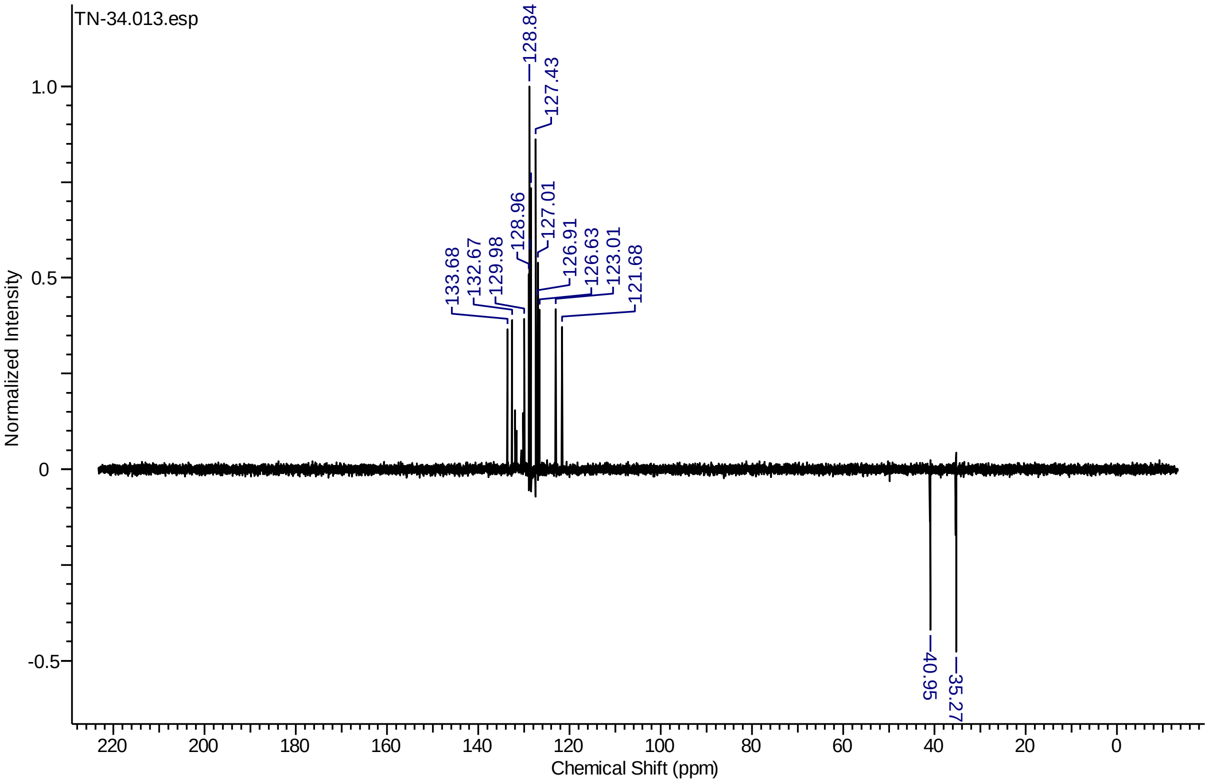

Figure S12: FT-IR spectrum of compound **4b**

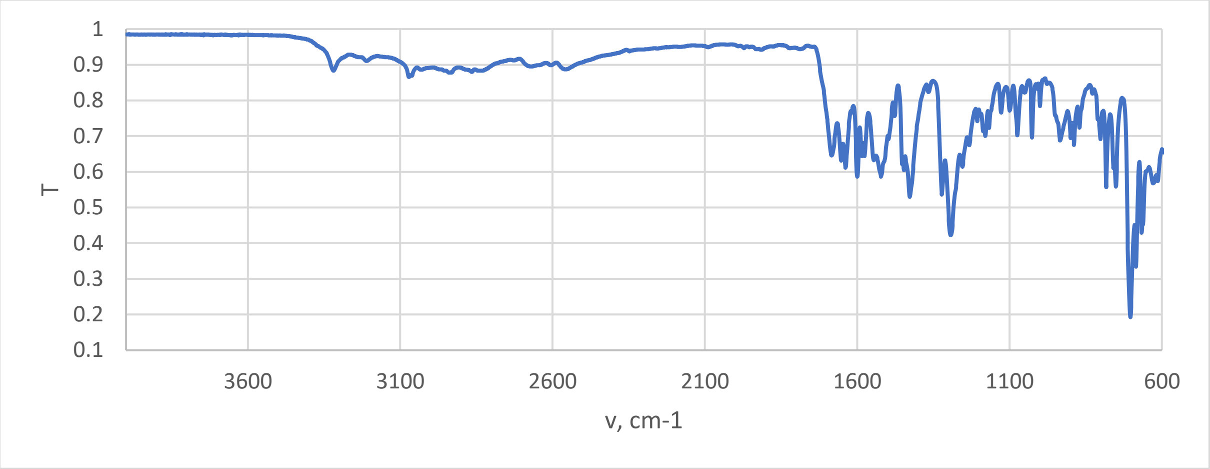

Figure S13: $^{1}$H-NMR spectrum of compound **4c**

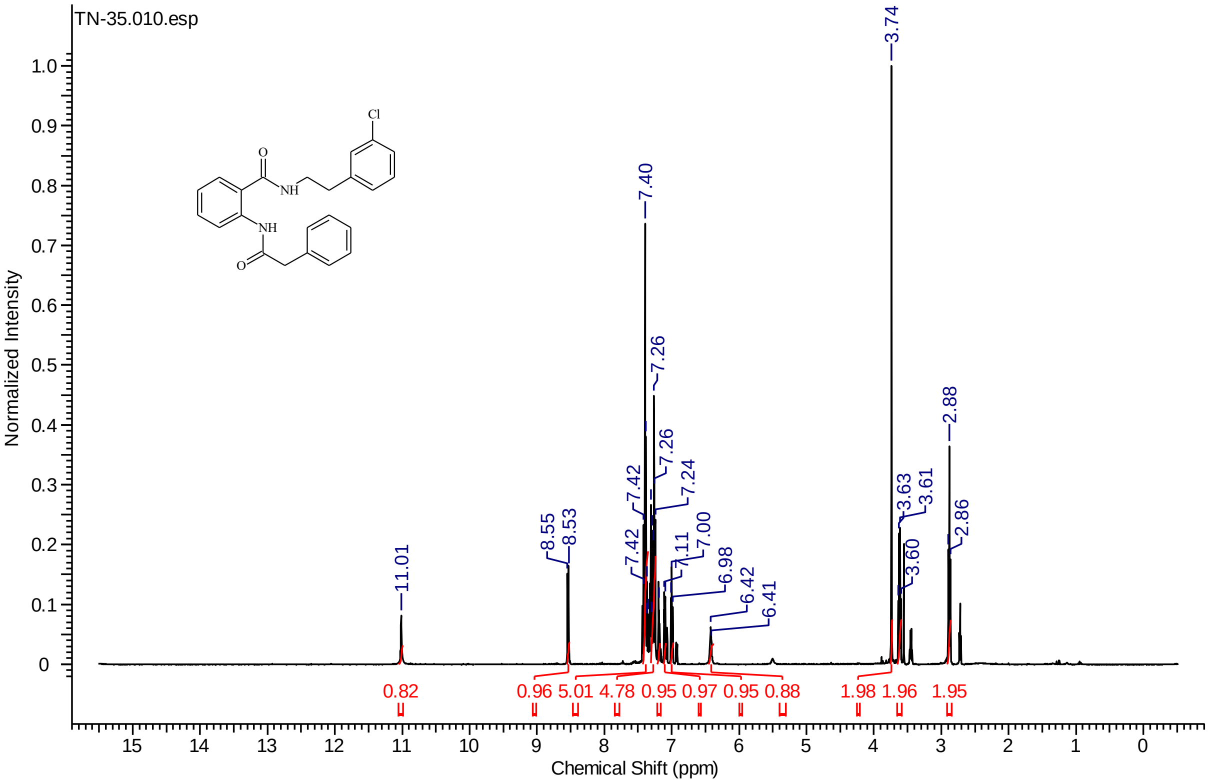

Figure S14: $^{13}$C-NMR spectrum of compound **4c**

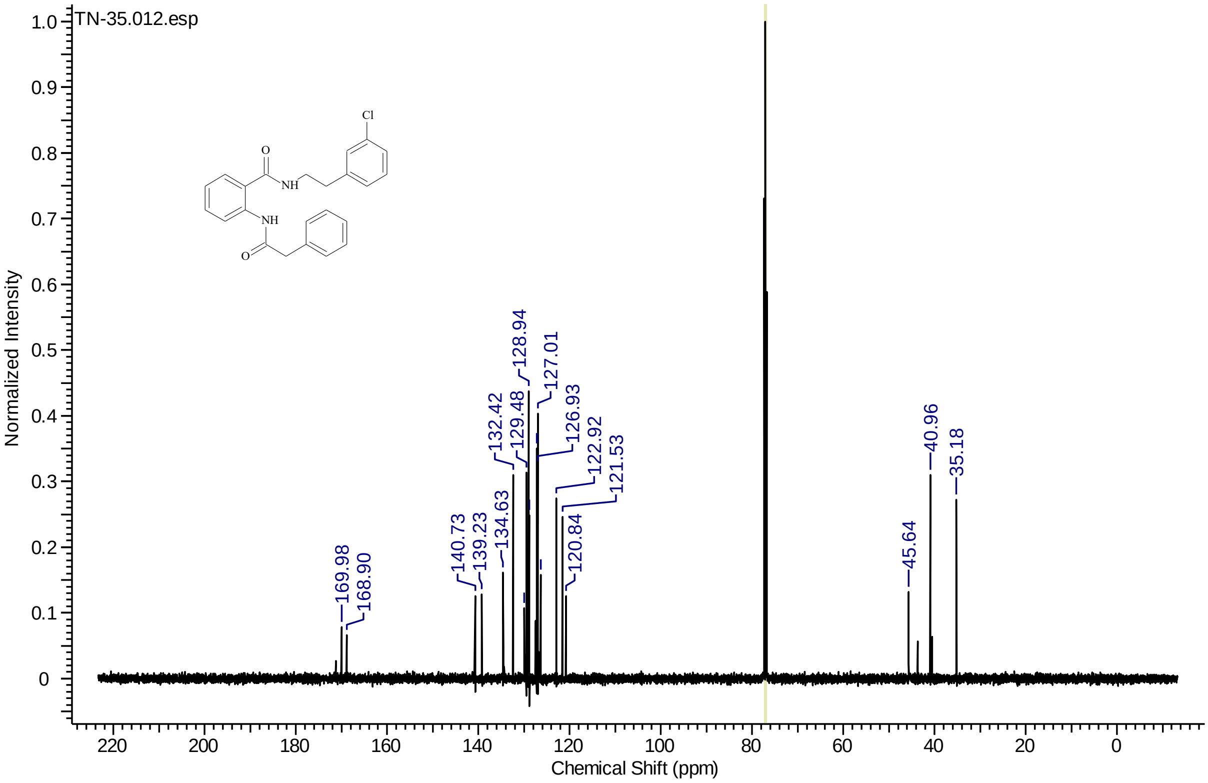

Figure S15: DEPT spectrum of compound **4c**

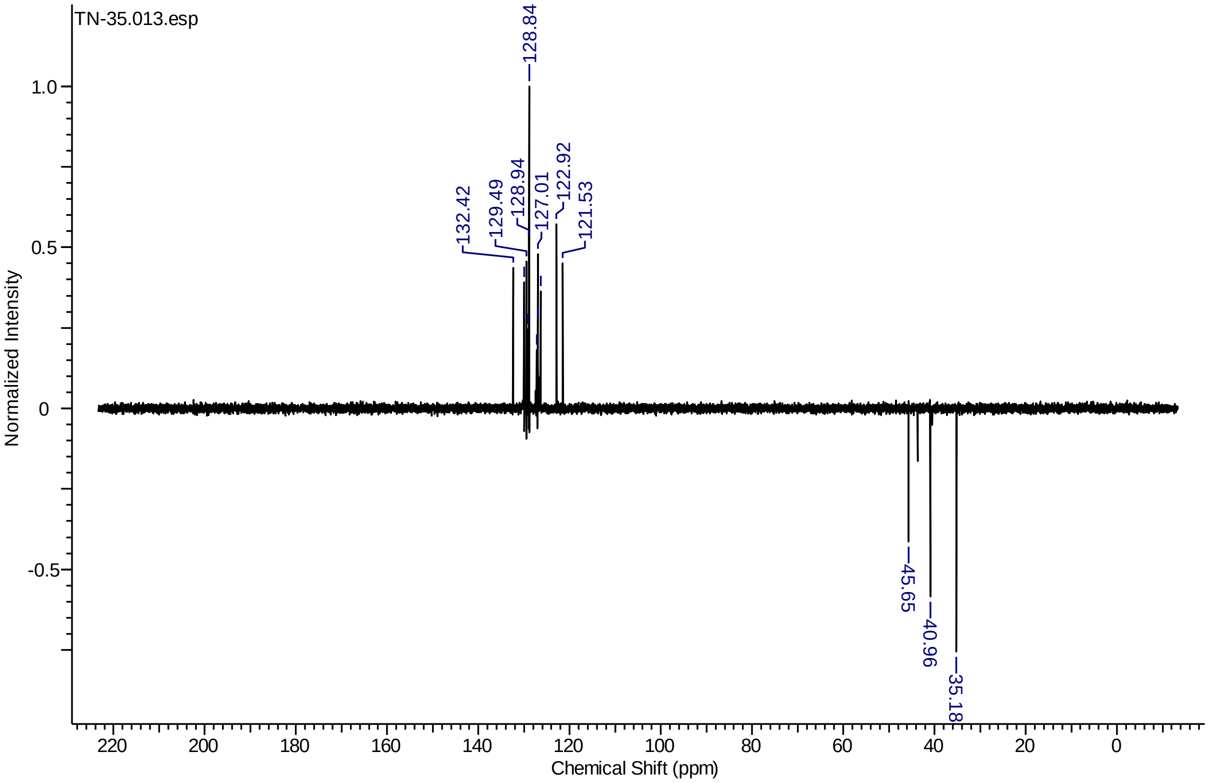

Figure S16: FT-IR spectrum of compound **4c**

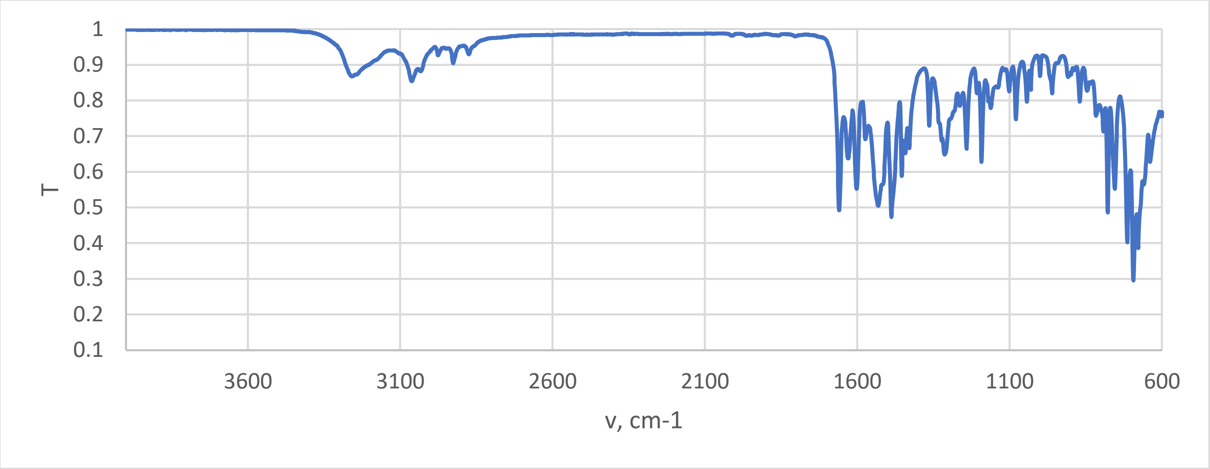

Figure S17: $^{1}$H-NMR spectrum of compound **4d**

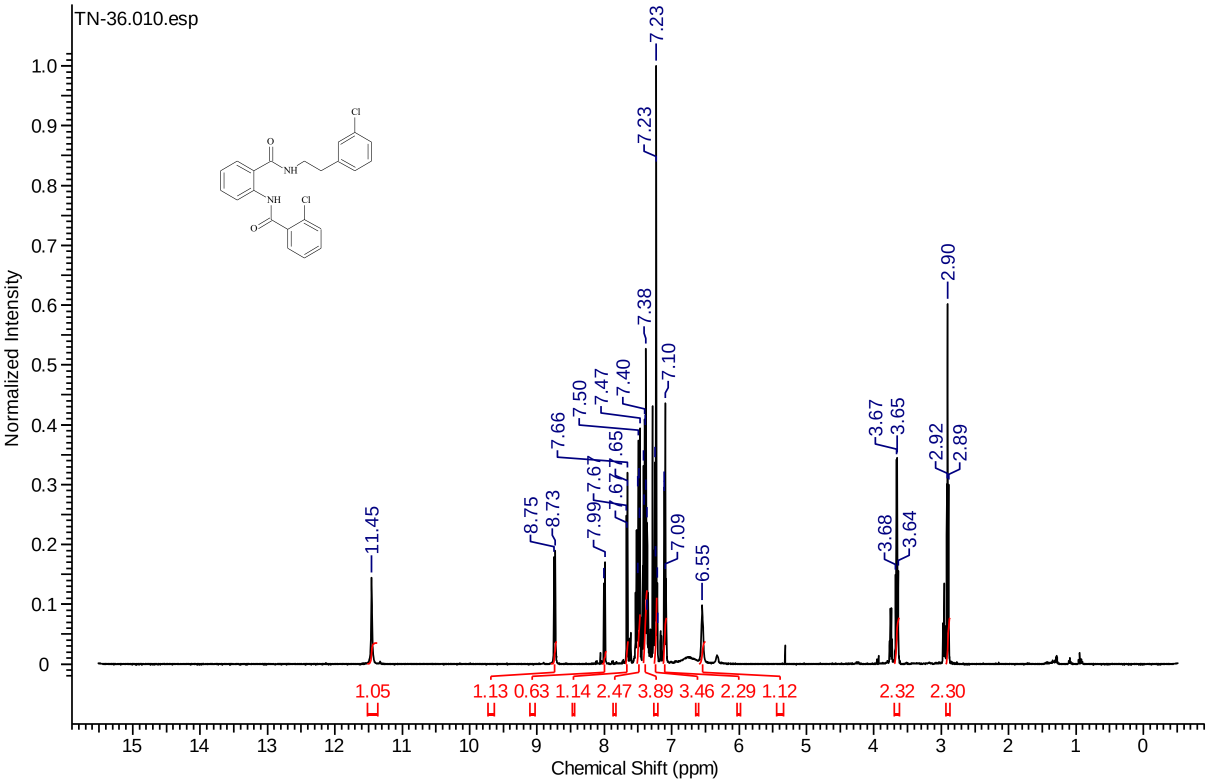

Figure S18: $^{13}$C-NMR spectrum of compound **4d**

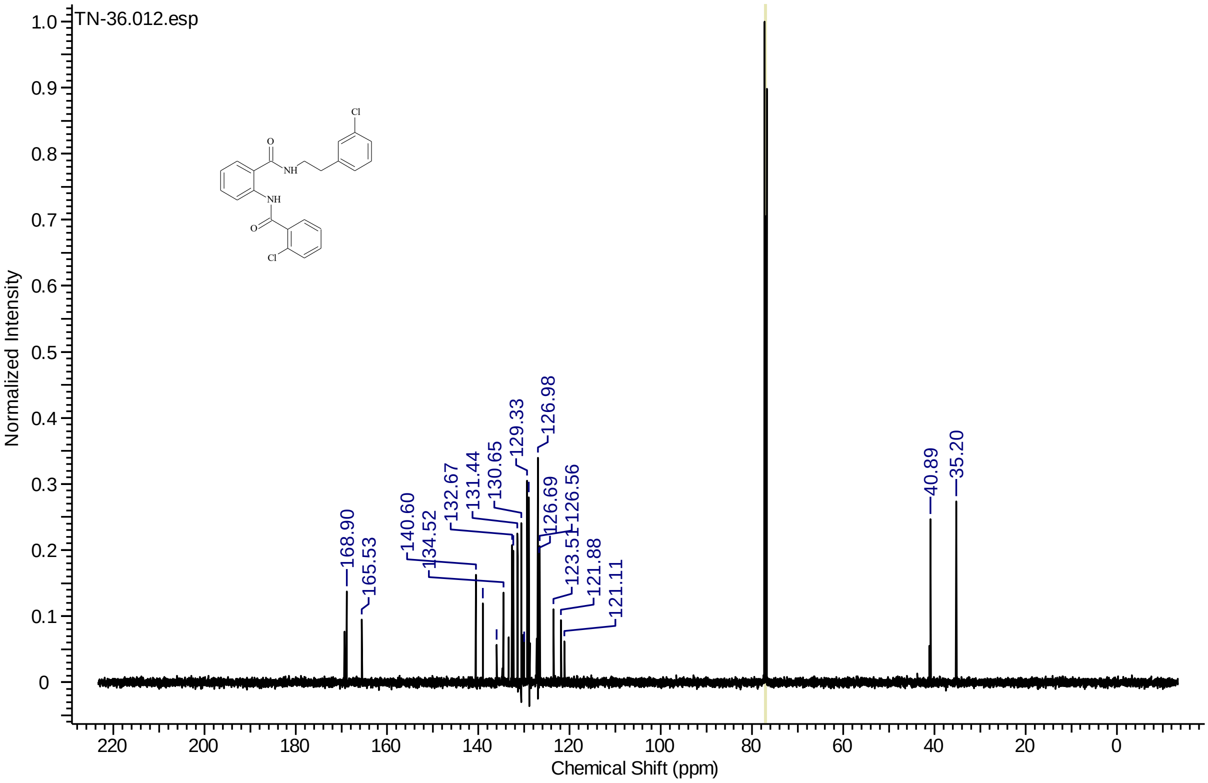

Figure S19: DEPT spectrum of compound **4d**

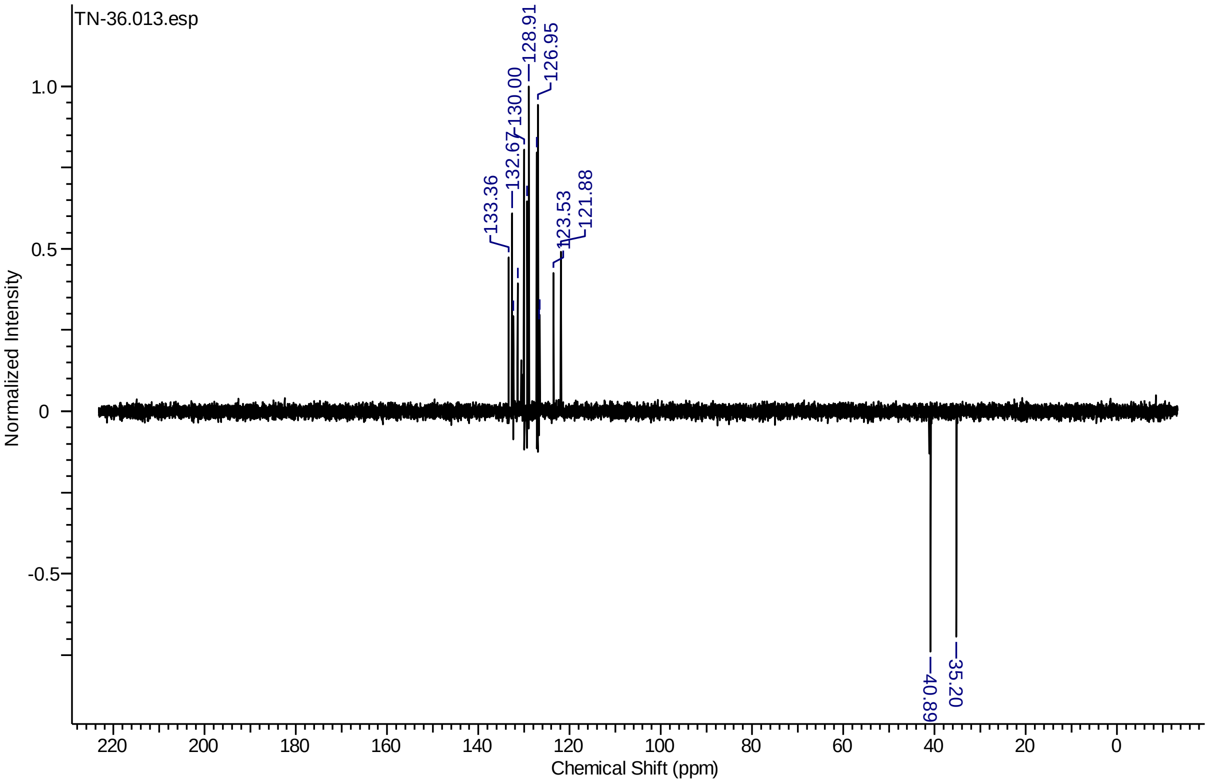

Figure S20: FT-IR spectrum of compound **4d**

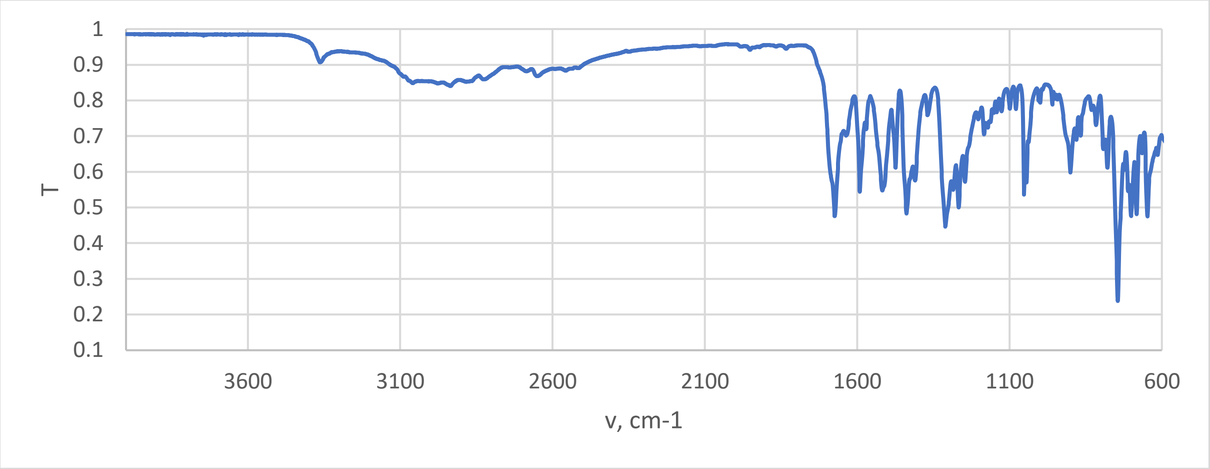

Figure S21: $^{1}$H-NMR spectrum of compound **4e**

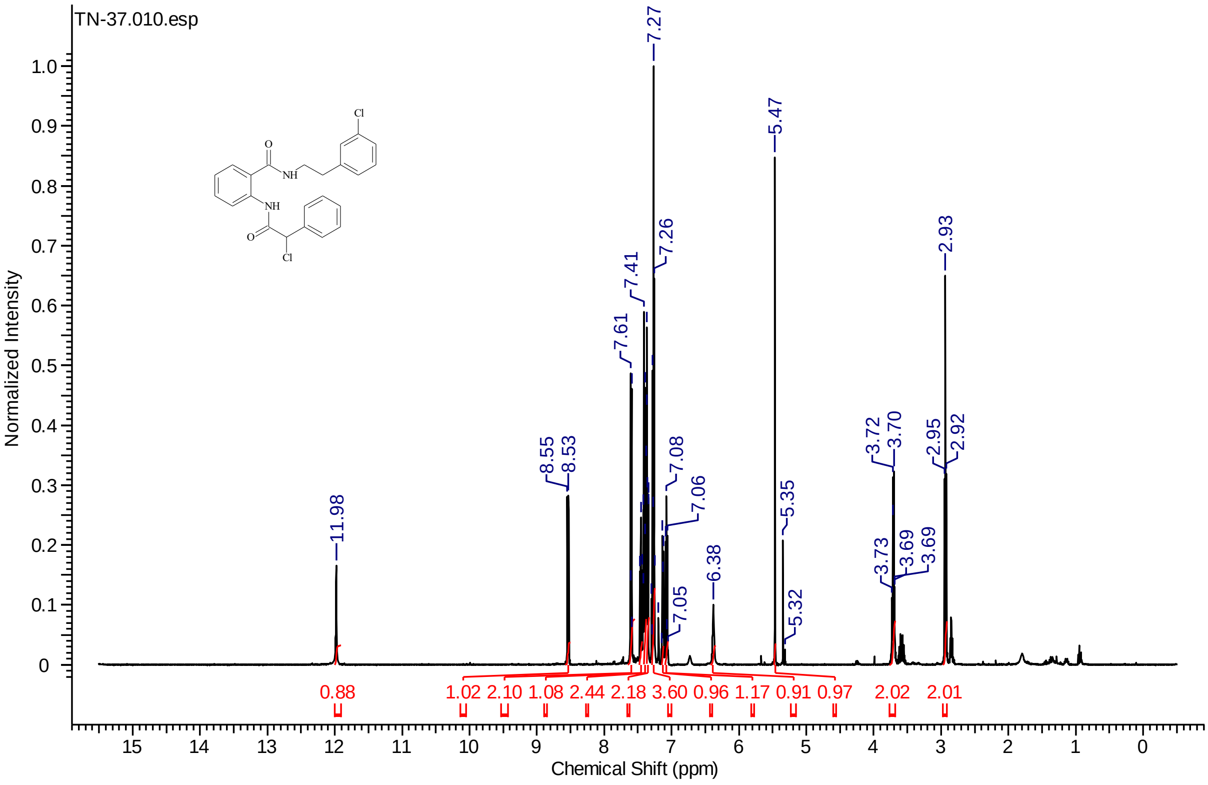

Figure S22: $^{13}$C-NMR spectrum of compound **4e**

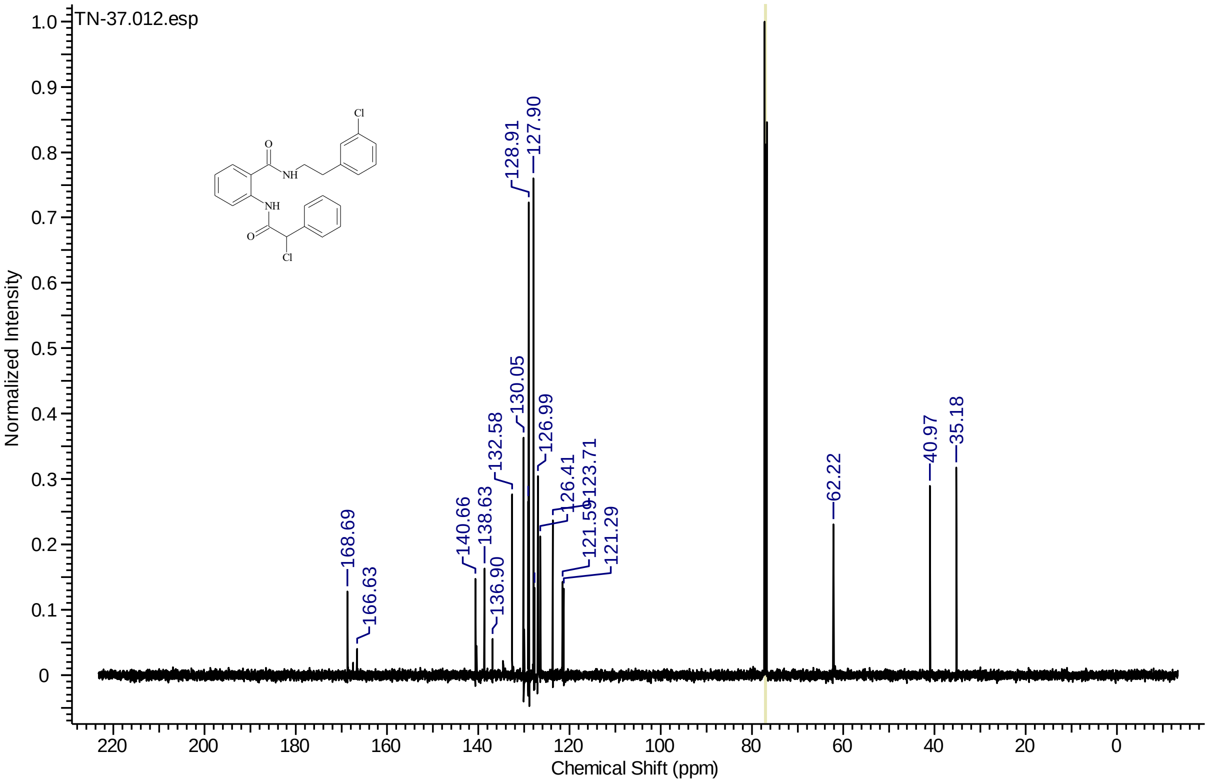

Figure S23: DEPT spectrum of compound **4e**

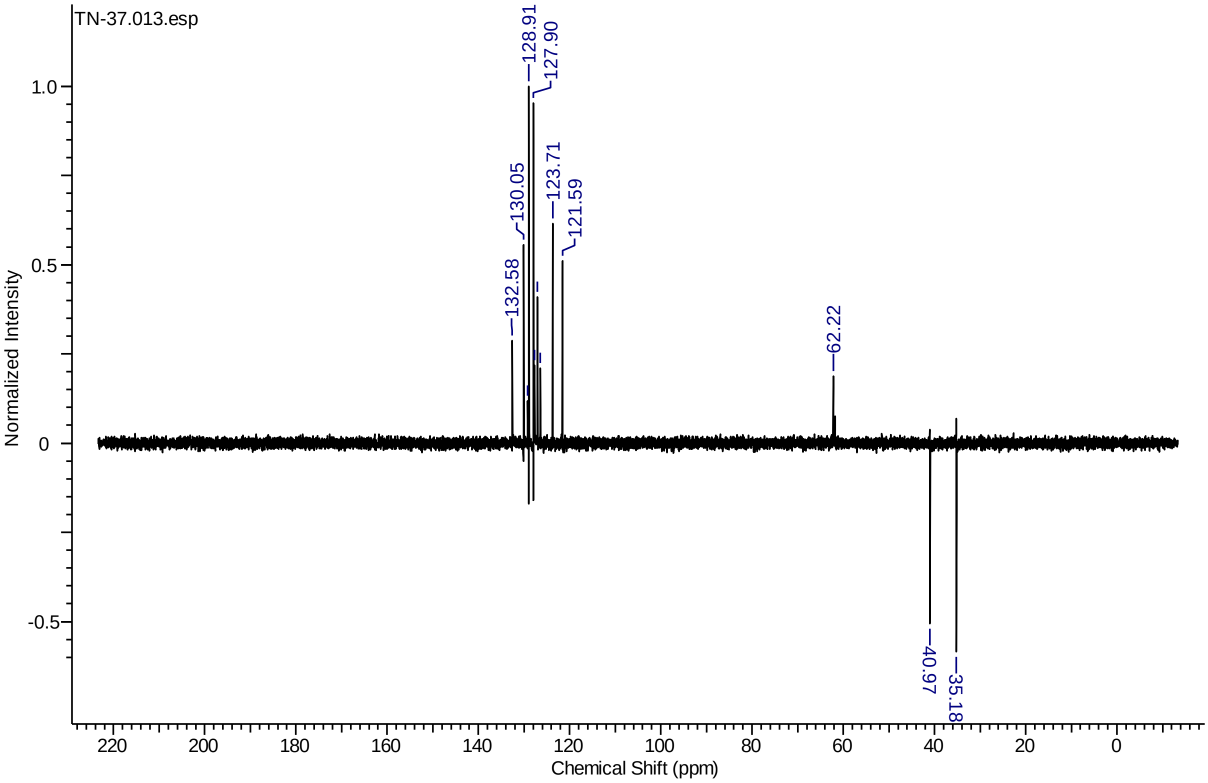

Figure S24: FT-IR spectrum of compound **4e**

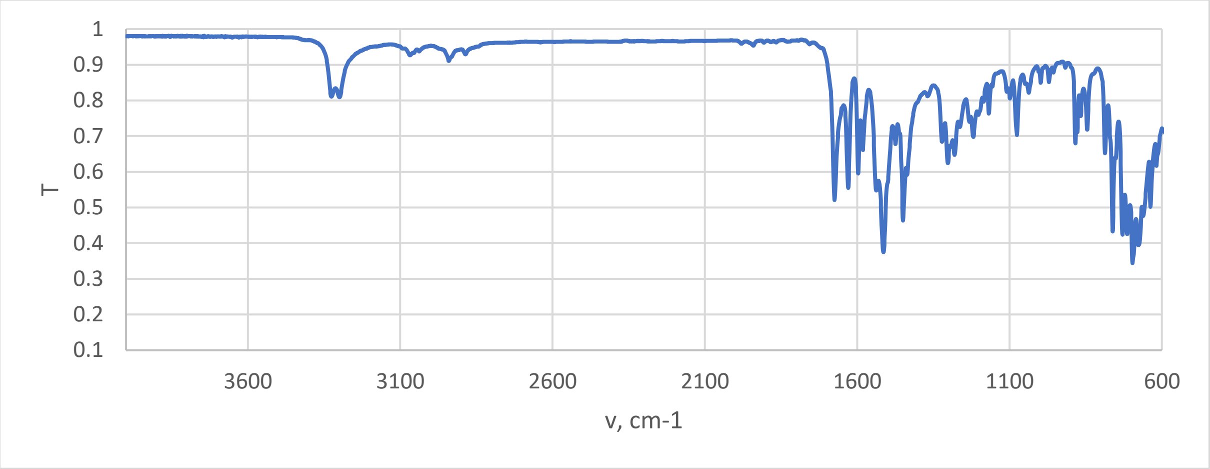

Supplement: Supplementary file 1 [file ijms-24-13855-s001.zip › ijms-2567042-supplementary.pdf]
